# An Electromechanical Power Law in Sustainable Thermally Drawn Triboelectric Nanocomposite Fibers for Sensing in Continuum Robots

**DOI:** 10.1021/acsami.6c00217

**Published:** 2026-04-01

**Authors:** Vishwa Pratap Singh, Nurbolat Issatayev, Syed Zubair Hussain, Yersaiyn Bushanov, Gulnur Kalimuldina, Mustafa Ordu

**Affiliations:** † UNAM - National Nanotechnology Research Center and Institute of Materials Science and Nanotechnology, 52948 Bilkent University, Ankara 06800, Türkiye; ‡ Department of Mechanical and Aerospace Engineering, School of Engineering and Digital Sciences, 214082 Nazarbayev University, Astana 010000, Kazakhstan

**Keywords:** Thermal drawing, Nanocomposites, Triboelectricity, SnO_2_, Continuum robotics

## Abstract

The development of self-powered and sustainable tactile sensors requires scalable materials that integrate electromechanical coupling, environmental compatibility, and high precision. Here, we report a universal electromechanical scaling law governing the voltage–force response in triboelectric nanogenerators (TENGs), established through sustainable SnO_2_ integrated polyvinylidene fluoride nanocomposite fibers fabricated via a thermal fiber drawing technique. The fibers exhibit enhanced crystallinity and interfacial polarization, yielding an open-circuit voltage of 37.2 V and a short-circuit current of 36.25 μA, with a corresponding peak power of 32.1 μW (243 mW m^–2^) under cyclic mechanical excitation. Beyond performance gains, the extracted force-dependent power law provides a transferable framework to benchmark and compare soft TENG fibers across loading conditions, addressing a major gap in standardized sensitivity metrics. Moreover, the resulting devices demonstrate long-term durability (>16,000 cycles) and exceptional sensitivity in robotic tactile and continuum actuation systems. Integration of the fibers into continuum robotic platforms enabled self-powered tactile sensing and rapid collision detection in free-space and in-pipe scenarios, achieving response times under 25 ms. This study establishes a physics-based framework for soft triboelectric systems, merging sustainable nanomaterials, scalable fiber processing, and universal electromechanical laws, paving the way toward self-powered, ecoconscious robotic and wearable interfaces.

## Graphical abstract

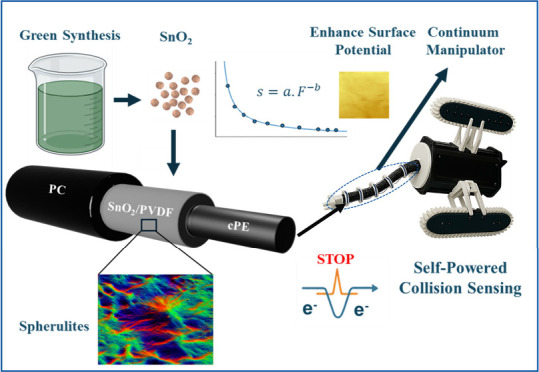

## 1 Introduction

Recent advancements in triboelectric nanogenerators (TENGs) have significantly influenced the landscape of future energy harvesting and sensing technologies.^1,2^ The persistent reliance on nonrenewable energy sources has led to severe economic and environmental challenges, thereby intensifying the demand for sustainable alternatives.^3,4^ Among various nanogenerator-based sensing mechanisms, namely piezoelectric (PENG),^5^ triboelectric (TENG),^6,7^ thermoelectric (TEG),^8,9^ and electromagnetic (EMG),^10^ TENGs stand out due to their simplicity, scalability, and high energy conversion efficiency derived from mechanical motion. Since their conceptual introduction in 2012,^11^ TENGs have garnered substantial research interest. Notably, given that approximately one-quarter of global energy consumption is attributable to tribological losses, the conversion of friction-induced mechanical energy into electrical energy presents a promising solution to the global energy crisis.^12^ Mechanical energy conversion in these systems is typically realized through contact, separation, or sliding interactions between materials possessing distinct polarities.^13^ The disparity in the electronegativity of the contact materials further contributes to charge transfer and enhances output performance.^14^ TENGs have demonstrated remarkable versatility across numerous application domains, including sensing^15^ and energy harvesting for self-powered devices in the fields of robotics,^16,17^ Internet of Things (IoT),^18^ artificial intelligence (AI),^16,19^ and machine learning (ML),^20,21^ as well as practical implementations in agriculture, biomedical devices, smart home systems, smart gadgets, motion detectors, and next-generation wearable electronics.^22^

In continuum robots, the ability to measure strain accurately is crucial for effective motion control. Several sensing strategies have been explored, including electromagnetic, Fiber Bragg Grating (FBG) sensors, and Hall effect sensors.^23–25^ FBGs offer high accuracy but come at a significant cost with elevated losses at large bending angles.^26^ Electromagnetic and Hall-based sensors are easily disturbed by external electromagnetic fields.^27,28^ To address these constraints, flexible electronic skins have been developed using conductive materials such as carbonaceous composites and liquid metals.^29,30^ However, the relatively low endurance of carbon-based structures and toxicity and leakage concerns in liquid metals for biomedical settings are major issues when utilizing these materials.^31,32^ Recently, polymer-based TENG tactile sensors offer a transformative solution to these challenges, particularly for minimally invasive and soft robotic applications.^20,33^ TENGs are inherently self-powered, significantly reducing electrical leakage risks in biomedical environments.^34^ Their flexibility allows seamless adaptation to the complex geometries of continuum robots.^35^ Although challenges like contact localization and signal crosstalk persist, advances in electrode design and signal processing continue to enhance their performance.^36^ Given their unique combination of safety, mechanical compatibility, and integration ease, TENG-based sensors offer a compelling direction for achieving full-body tactile perception in next-generation continuum robots.^37^

Among the common polymer-based triboelectric materials, polytetrafluoroethylene (PTFE), polyvinylidene fluoride (PVDF), fluorinated ethylene propylene (FEP), and polyimide (PI) as negative layers, and nylon, polydimethylsiloxane (PDMS), polyethylene terephthalate (PET), and poly­(methyl methacrylate) (PMMA) as positive layers, PVDF-based nanocomposites are the most suitable TENG materials due to their remarkable fluorine-based electronegativity and stable spontaneous polarization making it ferroelectric in nature.^38^ PVDF has five known phases: α, β, γ, δ, and ε; out of them the β-phase shows high polarity and γ-phase partial polarity, which further elevates the ferroelectricity of PVDF with increased electrical output.^39,40^ In the β-phase, PVDF chains adopt an all-trans (TTTT) conformation with uniformly aligned polar C–F bonds, giving a strong net dipole along the polymer backbone.^41,42^ β-Phase dominated PVDF has distinct regions of positive (H) and negative (F) potential, creating stronger interfacial coupling than the nonpolar α-phase dominated PVDF.^43,44^ The enhancement of the polar β-phase in PVDF-based polymers can be achieved by inducing molecular chain stretching through thermally driven elongation, such as in the thermal fiber drawing (TFD) process.^45,46^ The dielectric constant and ferroelectric behavior of the resulting structure can be further tuned by incorporating high-dielectric or ferroelectric nanofillers, including BaTiO_3_, ZnO, V_2_O_5_, which significantly improve triboelectric output due to the direct correlation between surface charge density and dielectric constant.^47^ Another widely adopted approach for performance enhancement is charge trapping, achieved by introducing functional nanomaterials such as reduced graphene oxide (rGO) and silver nanoparticles (AgNPs). These materials immobilize electrostatic charges at the interface, thereby strengthening polarization and improving stability.^48^

Beyond material engineering, surface modification techniques and electronegativity tuning also contribute to enhancing the charge transfer efficiency. Device geometry and architecture additionally play a critical role in optimizing triboelectric performance and expanding the applicability of TENGs.^49^ Among fabrication strategies, the TFD technique—originally developed for optical fiber processing—has gained attention due to its capability to produce complex, multicomponent fiber-based energy harvesters in a scalable manner.^50^ The process involves assembling a macroscale preform containing multiple functional materials, which is subsequently drawn into microscale fibers while preserving the original internal design.^51^ This method facilitates seamless integration of electrodes and active layers within a single continuous process, making it attractive for large-scale, textile-integrated TENG fabrication.^52^ Although thermal processing itself promotes the formation of polar crystalline phases in PVDF, challenges remain, particularly when incorporating nanomaterials into thermally drawn fibers due to the rheological constraints of multicomponent polymer systems. Nonetheless, successful demonstrations, such as PVDF-clad conductive polyethylene (cPE)-core patterned TENG fibers^53^ and thermally drawn SEBS-clad liquid-metal (EGaIn) core fibers,^54^ highlight the feasibility of this method for advanced fiber-based TENG design. In parallel, the emergence of green nanomaterial synthesis aligns with the sustainable vision of triboelectric systems. Unlike conventional chemical synthesis routes that often rely on hazardous precursors and energy-intensive processing, green synthesis uses biological extracts or natural reducing agents, offering a safer, low-cost, and scalable alternative. These bioderived nanostructures not only reduce the environmental impact but also enhance the charge-trapping ability and improve interfacial polarization, thereby contributing simultaneously to the functional and ecological advancement of next-generation TENG devices. Furthermore, despite rapid advances in TENGs for tactile sensing, the standardized quantification of their electromechanical performance remains a challenge. Sensitivity (*S*) is typically defined using a linear approximation, i.e., *S* = Δ*V*/Δ*F* (or Δ*V*/Δ*P*), which implicitly assumes a constant response across the operational range.^55^ However, this simplification fails to capture the intrinsic nonlinear behavior of soft, deformable systems, where contact area evolution and material compression do not scale linearly with the applied load. To address this, prior studies have employed piecewise linear fitting or contact-mechanics models derived from Hertzian theory.^56^ While effective for specific device geometries, these approaches remain configuration-dependent and do not provide a transferable electromechanical parameter for benchmarking force-sensitivity behavior across different TENG materials and fabrication strategies. Consequently, a universal framework to compare the intrinsic electromechanical response of fiber-based TENGs remains lacking.

In this study, green-synthesized SnO_2_ nanofillers are reinforced into PVDF to enhance its dielectric and triboelectric properties, as well as to promote the formation of the electroactive β-phase. The resulting polymer nanocomposite is used to fabricate TENG fibers through the TFD process, enabling scalable production. This process not only preserves the composite structure but also further enhances β-phase formation due to applied stress during fiber drawing. This resulting high-performance, flexible TENG fiber is designed to operate with three degrees of freedom suitable for continuum robots. The sensitivity of fibers is also characterized by the controlled force application by proposing a universal “Electromechanical Power Law” (*S*(*F*) = *a*·*F* ^–*b*^) that governs the voltage–force relationship in soft TENG fibers. We demonstrate that this law provides a more robust and physically meaningful framework than traditional linear models. Specifically, the intrinsic sensitivity coefficient (*a*) and decay exponent (*b*) allow for the first time a direct, transferable comparison of TENG performance across different force regimes and device morphologies. This approach shifts the focus from purely empirical observations to a standardized methodology for the soft electronics community.

## 2 Results and Discussion

### 2.1 Fabrication of TENG Fibers

Figure 1 illustrates the fabrication process of PVDF/SnO_2_ (PSO) nanocomposite films and fibers. The procedure includes synthesizing SnO_2_, preparing nanocomposite films, consolidating the films into a preform, and thermally drawing fibers spanning tens of meters from a 15-cm-long preform. PSO nanocomposite films with 0.5, 1.0, 2.0, 3.0, and 4.0 wt % SnO_2_ (hereafter referred to as PVDF/4.0%SnO_2_) were prepared by chemically dissolving the polymer PVDF, mixing it thoroughly with SnO_2_ under vigorous stirring, and drying the mixture in an inert environment, forming the nanocomposite films for the triboelectric active region. These nanocomposite films and additional polycarbonate (PC) films to assist the thermal drawing were wrapped around a conductive polyethylene (cPE) rod and consolidated to complete the preform.

**Figure 1:**
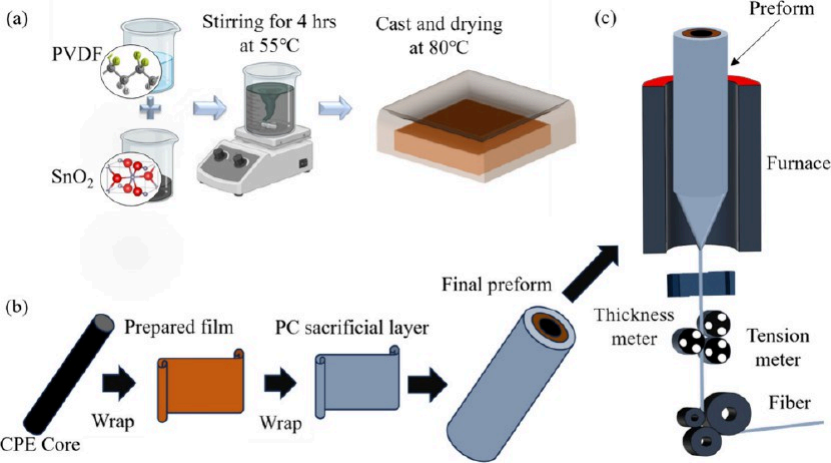
Fabrication of the polymer nanocomposite-based fibers. (a) Preparation of nanocomposite films, (b) development of preform, and (c) thermal fiber drawing process.

The final fiber dimensions depend on the preform’s shape/size, the feeding speed, the drawing speed, and the drawing temperature. The relationship between the diameters of the fiber and preform is given by

1 $$D_{\mathrm{fiber}}=D_{\mathrm{preform}}\cdot \frac{v_{\mathrm{feed}}}{v_{\mathrm{draw}}}$$

where *D*_fiber_, *D*_preform_, *v*_feed_ and *v*_draw_ are the diameter of fiber, diameter of preform, velocity of feeding, and velocity of drawing capstan, respectively. Multimaterial fibers were fabricated from the PVDF-based films under identical thermal drawing conditions for all preforms, namely, pristine PVDF, and PVDF with 0.5, 1.0, 2.0, 3.0, and 4.0% SnO_2_ nanofillers. Further loading of SnO_2_ led to the failure of the thermal fiber drawing process; therefore, the fibers were limited with a maximum 4% loading.

### 2.2 Materials Characterization

#### 2.2.1 Characterization of SnO_2_ Nanoparticles

The chemical composition and crystal structure of the green-synthesized SnO_2_ nanopowder were analyzed using X-ray diffraction (XRD) spectroscopy, as shown in Figure 2a. The XRD pattern reveals peaks at 26.6°, 33.9°, 37.9°, 51.8°, 54.7°, and 65.9°, corresponding to the (110), (101), (200), (211), (220), and (301) crystal planes, respectively. These diffraction peaks are well indexed to the tetragonal rutile phase of SnO_2_ (JCPDS card no. 41-1445), confirming the formation of crystalline SnO_2_ with a *P*4_2_/*mnm* space group structure. The schematic was developed by using the XRD details via the POSCAR VESTA software in Figure 2e. Additionally, the elemental composition and chemical bonds in the nanopowder were examined using X-ray photoelectron spectroscopy (XPS) analysis. Figure 2b shows that the SnO_2_ powder exhibits characteristic peaks for elements C, O, and Sn. The presence of the C peak can be explained by air absorption on the surface of the sample. The high-resolution spectrum of Sn3d (3d_5/2_ and 3d_3/2_) in the nanopowder (Figure 2c) shows peaks at binding energies of approximately 487.51 and 495.85 eV, corresponding to Sn^4+^. The microstructure of the sample was further analyzed by using transmission electron microscopy (TEM), as presented in Figure 2d. The TEM image reveals the formation of SnO_2_ nanopowders with sizes ranging from 4 to 10 nm.

**Figure 2:**
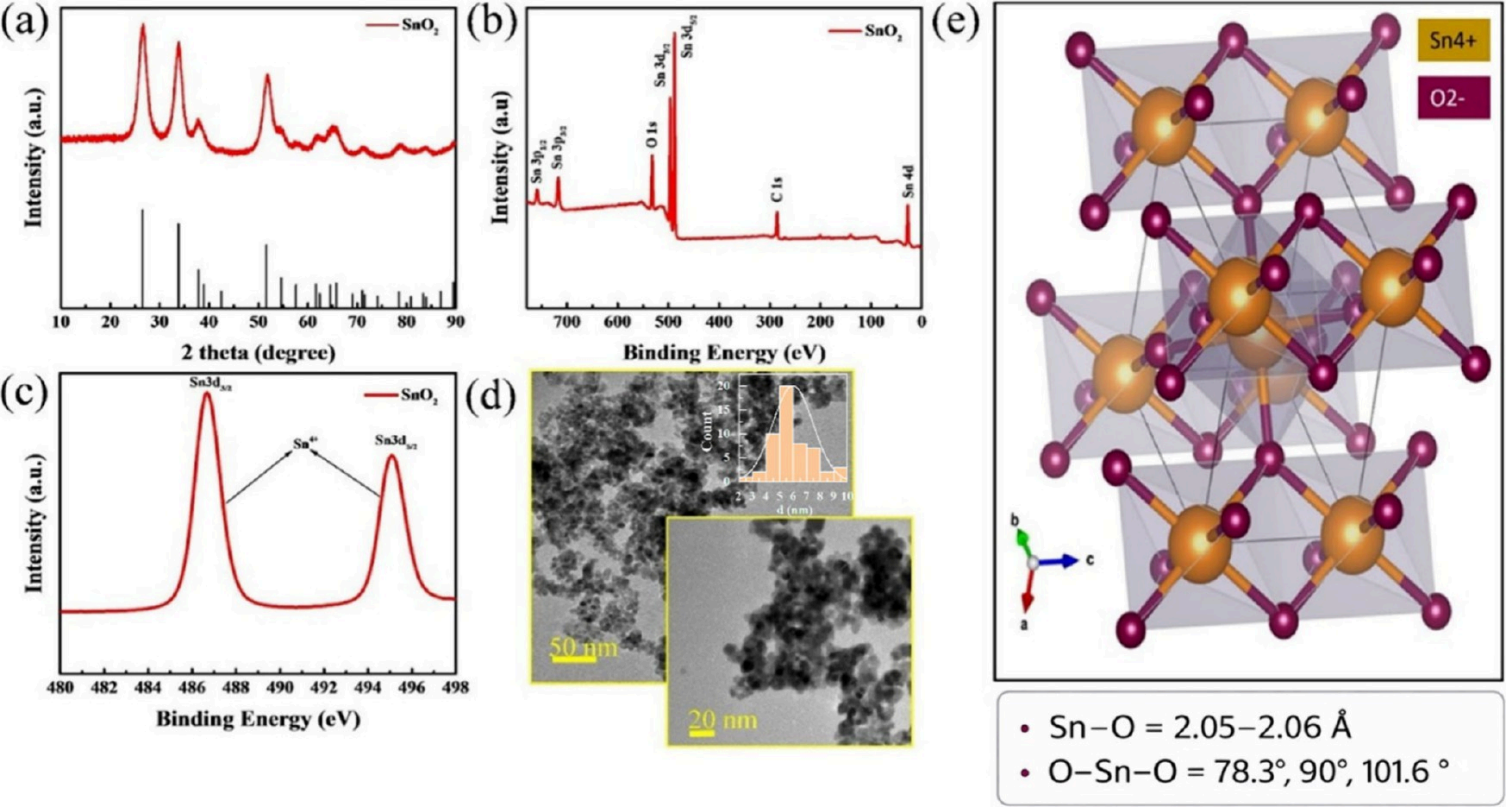
Material characterization of the green-synthesized SnO_2_ nanopowder. (a) XRD pattern, (b) XPS survey, (c) high-resolution XPS spectra of Sn4d, and (d) TEM images of SnO_2_ with histogram. (e) Schematic of SnO_2_ structure developed from XRD data using VESTA software.

#### 2.2.2 Morphological Characterization of TENG Fibers

Figure 3a shows a bundle sample of 5 m long drawn PSO nanocomposite fiber, demonstrating the scalability of the method. Figure 3b provides the SEM-based elemental dot mapping of the surface of the nanocomposite fiber with a homogeneous distribution of SnO_2_ nanofillers inside the polymer matrix, even with 4.0% reinforcement. Figure 3c shows the angled cross section of fiber having an active layer of PSO nanocomposite and cPE as the electrode core enclosed by the PC sacrificial layer; the overall diameter of the fiber is 1.30 ± 0.01 mm and 0.9 ± 0.01 mm without the PC layer. Zooming in further on the active triboelectric layer reveals a pronounced presence of spherulitic structures with radial symmetry and a lamellar substructure ranging from 2 to 6 μm in diameter. Such morphology under thermal drawing conditions, which typically involve chain disentanglement, shear-induced flow, and temperatures above the crystalline melting point, is fascinating. The spherulite formation is not only retained but appears to be enhanced with increasing SnO_2_ nanofiller content (0.5–4.0%), indicating an active nucleation–recrystallization process driven by nanofiller–polymer interactions under dynamic thermal and mechanical fields. The inset of Figure 3c demonstrates the spherulite, and then further Figure 3d shows the variation of the size and numbers of the spherulite in the polymer nanocomposites along with the nanofillers. The appearance of these spherulitic domains can be rationalized using heterogeneous nucleation theory,^57^ where the energetic barrier for nucleation Δ*G*_het_^*^ is lowered by the presence of foreign surfaces (SnO_2_), as described

2 $$\Delta G_{\mathrm{het}}^{*}=\Delta G_{\mathrm{hom}}^{*}f\left(\theta \right)\;\mathrm{with}\;\Delta G_{\mathrm{hom}}^{*}=\frac{16\;\pi \gamma^{3}}{3\;\Delta G_{v}^{\;2}}$$

Here, γ is the surface free energy of the crystal–melt interface, *ΔG*_v_ is the volumetric free energy change during crystallization, and *f*(*θ*) is a geometrical factor that accounts for the wetting angle between the polymer and nanofiller surface. Since SnO_2_ provides high surface energy contrast and polar interactions with the fluorinated PVDF matrix, the function *f*(*θ*) leads to a significant reduction in nucleation barrier. In thermal drawing, the local undercooling, Δ*T* = *T*_m_ – *T*, near the solidifying fiber boundary layer increases due to rapid cooling and convective heat removal. This enhances the thermodynamic driving force *ΔG*_v_, thus further lowering *ΔG*_v_^*^ and supporting rapid nucleation and subsequent radial growth. The Hoffman–Lauritzen theory governs the kinetics of lamellar growth from the melt.^58^ The spherulite growth rate *G* is determined by secondary nucleation on the lateral surfaces of preexisting lamellae given as

3 $$G=G_{0}\;\exp\left(-\frac{U^{*}}{R\left(T-T_{\infty }\right)}\right)\;\exp\left(-\frac{K_{g}}{T\;\Delta T}\right)$$

where *G*_0_ is a pre-exponential factor, U* is the activation energy for segmental motion across the crystal interface, *R* is the universal gas constant, *T*_∞_ is the hypothetical temperature below which all motion ceases, *K*_g_ depends on the surface free energies of chain folds and lateral faces.

**Figure 3:**
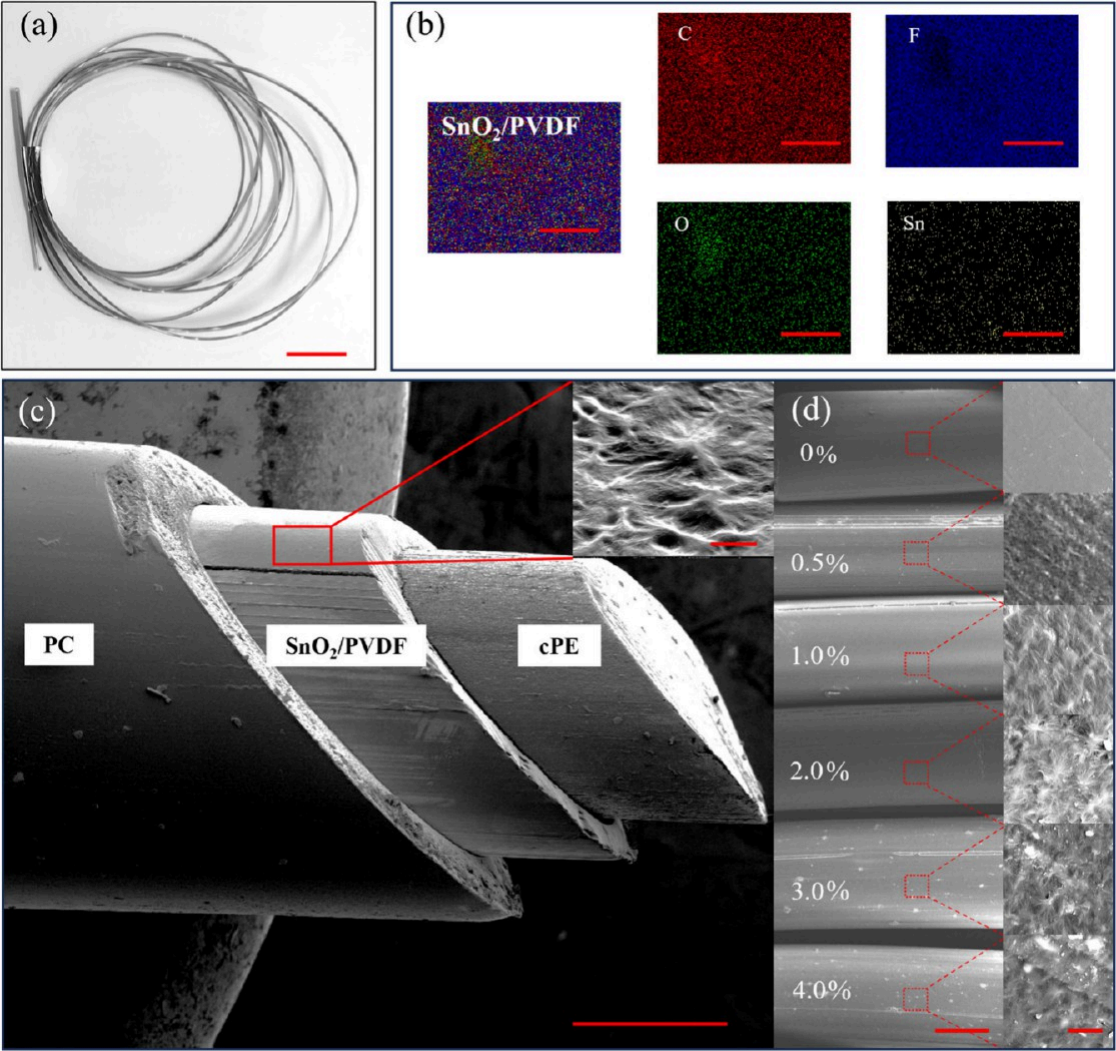
Elemental composition and surface properties of fabricated PSO fibers. (a) Fiber bundle (scale bar: 1 cm), (b) elemental mapping of layer of 4.0% PSO fiber (scale bar: 50 μm). (c) SEM cross-sectional image showing the multilayer structure of the fiber consisting of PC cladding, PSO active layer, and cPE electrode (scale bar: 400 μm), with inset highlighting the surface morphology demonstrating the spherulite formation (scale bar: 2 μm). (d) SEM images of surfaces of fibers with varying SnO_2_ reinforcement (0–4.0%) (scale bar: 500 μm), showing microstructural evolution in insets (scale bar: 10 μm).

Despite drawing at 254 °C well above PVDF’s melting temperature (∼177 °C), the extensional flow aligns molecular chains, while high thermal gradients at the fiber boundary re-establish favorable undercooling. The synergistic interaction of chain orientation, SnO_2_-induced heterogeneous nucleation, and rapid heat dissipation locally promotes the reformation of lamellar stacks with radial symmetry, characteristic of spherulites. Spherulitic growth is further governed by self-generated impurity and pressure gradients in the crystallizing melt.^59^ The relevant diffusion length δ, which represents the spatial extent of these fields, is given by

4 $$\delta =\frac{D}{G}$$

where *D* is the diffusivity of polymer chains or impurities in the melt, and *G* is the growth rate of the lamellae. At higher nanofiller loadings, chain mobility near the interface is reduced, leading to a smaller *D* and smaller δ, i.e., a more confined diffusion gradient. This confinement results in tighter, more closely spaced lamellae, and, thus, denser spherulitic populations. Additionally, kinetic interface instability may contribute to tip splitting and noncrystallographic branching, which is critical to maintaining radial space-filling growth at elevated draw speeds.^59,60^

#### 2.2.3 Structural Characterization of TENG Fibers

The crystal structure and phase analysis of the fiber material composition were conducted using XRD and Fourier transform infrared spectroscopy (FTIR) techniques, as shown in Figure 4. These methods were employed to determine the crystalline phases and quantify the electroactive phase content. XRD measurements were carried out on both PVDF films and their corresponding fibers to analyze the crystalline phase distribution. The diffraction pattern of the PVDF film shows distinct peaks at 2θ = 17.7°, 18.3°, 19.9°, and 26.5°, which are indexed to the (100), (020), (110), and (021) planes of the α-phase, respectively (Figure S2b, ).^61,62^ After fiber drawing, the diffraction peaks corresponding to the α-phase at 17.7°, 18.3°, and 26.5° diminish in intensity, indicating suppression of the α-phase, as shown in Figure 4a. This reduction becomes progressively stronger with increasing SnO_2_ content, suggesting that both the drawing process and the incorporation of SnO_2_ nanofillers facilitate the α to β phase transformation in PVDF. Notably, the 19.9° α-phase peak is replaced by a new peak at 20.6°, associated with the (110)/(200) planes of the β-phase. To further explore the phase transformation, FTIR was performed. The FTIR spectrum of the PVDF film () shows prominent α-phase peaks at 763 cm^–1^, 795 cm^–1^, 854 cm^–1^, and 870 cm^–1^, with weak β-phase peaks at 510 cm^–1^, 840 cm^–1^, and 1234 cm^–1^, and γ-phase peaks at 833 cm^–1^ and 1279 cm^–1^. After thermal drawing, the fibers show a marked decrease in the intensity of α-phase peaks, while β-phase peaks at 510 cm^–1^ and 840 cm^–1^ become more pronounced, increasing with higher SnO_2_ loading. A new β-phase peak emerges at 1401 cm^–1^.^63^ Although some γ-phase peaks overlap with those of the β-phase, the absence of shoulder peaks at 833 cm^–1^ and 1234 cm^–1^ suggests a dominant presence of the β-phase, shown in Figure 4b. The absorbance peaks at 764 cm^–1^ (*A*_α_) and 840 cm^–1^ (*A*_β_) were used to identify the percentages of α and β phases, respectively.^60^ The relative fraction of the β-phase, *F*(*β*), was calculated using the following expression based on the Lambert–Beer law

5 $$F\left(\beta \right)=\frac{A_{\beta }}{\left(K_{\alpha }/K_{\beta }\right)A_{\alpha }+A_{\beta }}$$

where *A*_α_ and *A*_β_ are the absorbance values at 763 cm^–1^ and 840 cm^–1^, and *K*_α_ = 6.1 × 10^4^, *K*_β_ = 7.7 × 10^4^ are their respective absorption coefficients. The β-phase content increased from 29.7% in the PVDF film to 38.3% in thermally drawn PVDF fibers, as shown in Figure 4d, reaching up to 46.2% with the incorporation of 4.0% SnO_2_. These results indicate that the thermal drawing process promotes α to β phase transition, driven by mechanical stretching and thermal energy. The presence of SnO_2_ further enhances β-phase formation, likely due to strong interactions between the nanofiller and the PVDF matrix, which facilitate the nucleation of the electroactive β-phase. Although the α-phase is not eliminated completely, the coexistence of α and β phases in the fibers suggest partial transformation. The enhanced β-phase fraction, attributed to both thermal drawing and SnO_2_-induced nucleation, contributes to an increased interfacial polarization and improved molecular orientation, essential for the electroactive performance of PVDF nanocomposites.

**Figure 4:**
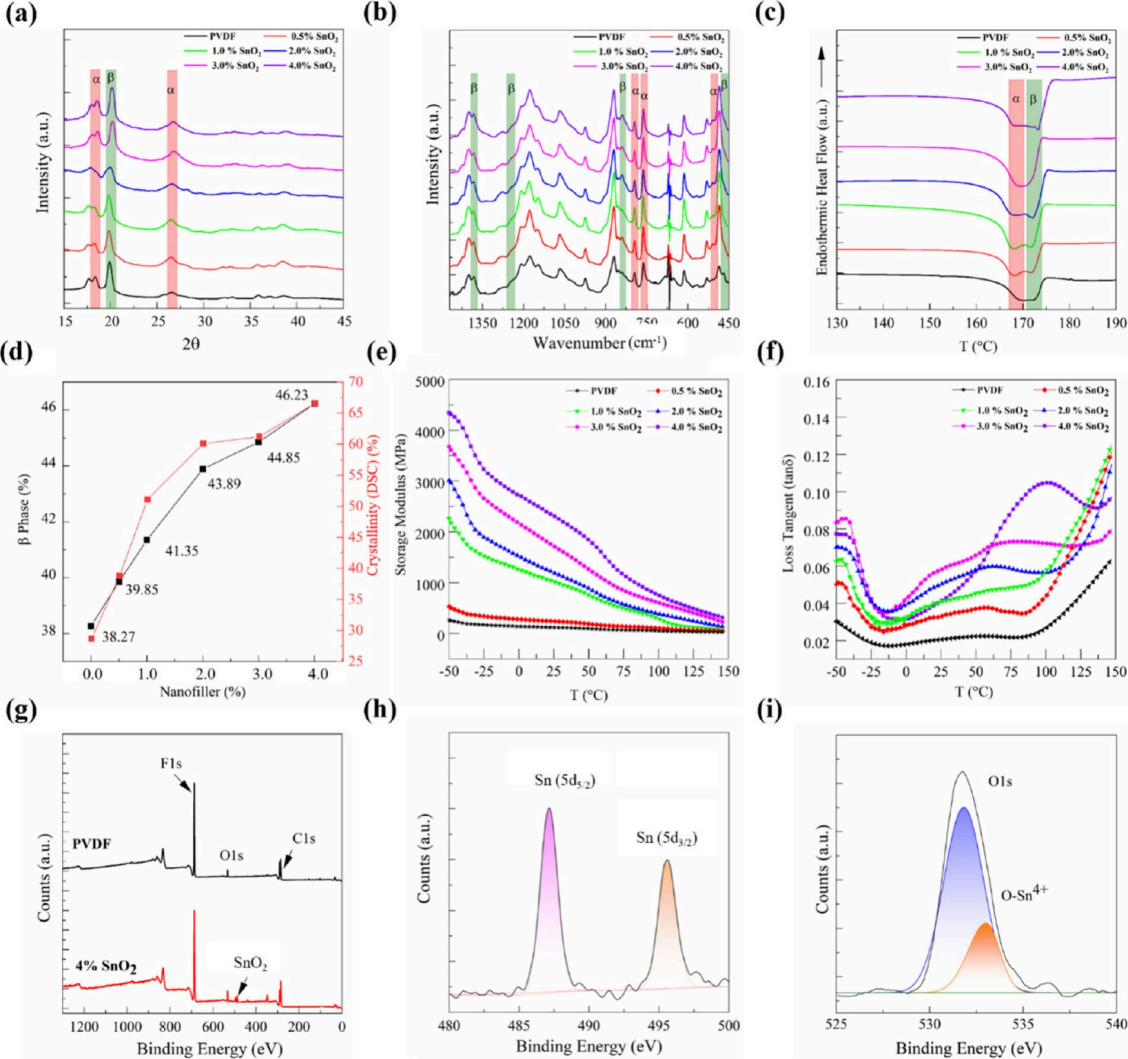
Structural, thermal, dielectric, and surface chemical analysis of pristine PVDF and PSO nanocomposites with varying filler concentrations. (a) XRD patterns showing the increase of the β-phase with SnO_2_ incorporation. (b) FTIR spectra confirming the phase transitions and increased β-phase fraction with nanofiller concentration. (c) DSC thermograms illustrate changes in melting behavior and crystallinity with nanofiller content. (d) Variation of β-phase content and crystalline with nanofiller loading. (e) Storage modulus and (f) dielectric loss tangent as a function of temperature. (g) XPS survey spectra of PVDF and 4.0% PSO composite. (h) High-resolution XPS spectrum of Sn3d and (i) O 1s, confirming the presence and oxidation state of SnO_2_ within the polymer matrix.

The thermal behavior of pristine PVDF and SnO_2_-filled composites was examined by differential scanning calorimetry (DSC), as shown in Figure 4c. The degree of crystallinity *X*_c_ was determined from the melting enthalpy given by

6 $$X_{c}=\frac{\Delta H_{f}\left(1-\phi \right)}{\Delta H_{f}^{o}}\times 100$$

where Δ*H*_f_ is the measured enthalpy of fusion of the sample, Δ*H*_f_^o^ is the enthalpy of fusion for 100% crystalline PVDF (103.40 J/g), and ϕ is the weight fraction of SnO_2_ in the polymer matrix. Due to enhanced dipole–dipole interactions, the electroactive β-phase exhibits a higher coherence energy, resulting in a lower endothermic melting temperature (*T*_m_) for the α-phase compared to the β-phase. As shown in Figure 4c, a shift in *T*_m_ indicates an increased proportion of the electroactive phase.

Dynamic mechanical analysis (DMA) was used to measure viscoelastic behavior of pristine PVDF and its SnO_2_-reinforced composite films to rationalize the processing limitations encountered during thermal drawing with higher filler loading. The storage modulus (*G*″) profiles in Figure 4e reveal a strong reinforcement effect with increasing filler content, where *G*′ at −50 °C rises by almost an order of magnitude (4.3 GPa) for 4.0% SnO_2_ relative to neat PVDF (0.28 GPa). This pronounced stiffening originates not only from the filler network and immobilization of PVDF chains at the polymer–filler interface but also from the nucleating action of SnO_2_, which promotes higher crystallinity and stabilizes electroactive crystalline domains correlating with the DSC measurement. The elevated *G*′ values at subambient temperatures and the slower modulus decay on heating indicate a larger fraction of crystalline lamellae that act as physical cross-links, corroborating the filler-induced crystallization effect.^58^ Moreover, the constrained chain mobility near SnO_2_ surfaces favors the transformation of PVDF chains into the polar β-phase upon stretching, which is consistent with the enhanced DSC and FTIR analyses observed in composite systems. The pronounced drop in *G*′ near the glass transition reflects the onset of cooperative segmental motion, whereas the convergence of modulus values at high temperature (>120 °C) indicates matrix softening where filler effects are diminished. Complementary insights are obtained from the tan δ curves, which display a minimum around −25 °C (low damping, frozen local relaxations) and a broad midtemperature hump (20–100 °C) that intensifies with SnO_2_ loading. This hump is characteristic of interfacial and constrained-amorphous relaxations, providing indirect evidence of enhanced crystalline–amorphous interphase regions introduced by the fillers. Finally, the sharp rise in tan δ above 120 °C corresponds to lamellar softening and incipient melting, which defines the upper limit of the workable processing range. Collectively, these findings confirm that SnO_2_ incorporation simultaneously improves crystallinity and promotes β-phase formation, thereby enhancing electroactive functionality, but excessive reinforcement (>4.0%) leads to an unfavorable balance of stiffness and damping, severely limiting the fiber drawing ability.

To investigate the elemental composition and chemical interactions in the composite system, XPS was performed on pristine PVDF and nanocomposite PVDF incorporating 4.0% SnO_2_, as demonstrated in Figure 4g–i. The survey spectra confirmed the presence of C, F, and O elements in both samples with distinct Sn signals emerging only in the composite, indicating successful incorporation of SnO_2_. High-resolution F 1s spectra revealed a slight shift in the binding energy from 687.75 eV in pristine PVDF to 687.45 eV in the SnO_2_-loaded sample, suggesting intermolecular interactions between the electronegative fluorine of PVDF and the SnO_2_ surface. The C 1s spectra further supported this interaction, with the −CH_2_ and −CF_2_ peaks observed at 286.6 and 290.9 eV in PVDF, respectively, exhibiting minor shifts to higher binding energies in the composite due to the presence of oxygenated carbon species, likely originating from SnO_2_ surfaces.^61^ The O1s spectrum in the composite displayed a dominant peak at 531.5 eV, attributed to lattice oxygen in SnO_2_ (O–Sn^4+^), and a smaller shoulder indicating surface hydroxyl groups. Furthermore, the Sn3d spectrum exhibited distinct peaks at 487.3 eV (Sn3d_5/2_) and 495.7 eV (Sn3d_3/2_), confirming the presence of Sn^4+^ species. These observations collectively confirm the successful dispersion of SnO_2_ within the PVDF matrix and highlight the interfacial chemical interactions that may contribute to the altered surface polarity and enhanced functional properties of the nanocomposite.

#### 2.2.4 Surface Potential Measurement and Electrostatic Finite Elemental Modeling

The surface potential and work function evolution of pristine PVDF and 4.0% PSO composite films were examined by Kelvin probe force microscopy (KPFM) using a Pt/Ir-coated conductive tip (work function = 5.2 eV) calibrated against a gold reference. The contact potential difference (CPD) was recorded at a lift height of 100 nm to minimize electrostatic artifacts, as shown in Figure 5a. The mean CPD values obtained were −0.15 ± 0.02 V for pristine PVDF and −1.12 ± 0.05 V for 4.0 wt % PSO, corresponding to calculated work functions of 5.35 ± 0.02 eV and 6.37 ± 0.05 eV, respectively. The work function of the polymer films was calculated from the CPD according to^64^

7 $$\Phi_{sample}=\Phi_{tip}-eV_{CPD}$$

where Φ_sample_ is the sample work function, Φ_tip_ is the calibrated tip work function, *e* is the elementary charge, and *V*_CPD_ is the average contact potential difference between the tip and the sample surface. This substantial increase (≈1.05 eV) in the work function of the PSO nanocomposite reveals strong electronic coupling between SnO_2_ and the PVDF matrix. The enhancement arises from the higher electron affinity of SnO_2_ (**∼**4.5 eV) relative to pristine PVDF (∼2.6 eV), which drives electron transfer from PVDF chains toward the SnO_2_ nanofillers until Fermi level equilibration is reached. This charge redistribution deepens the interfacial potential well and increases the surface potential of the composite, as schematically illustrated in Figure 5b, where SnO_2_ acts as an electron sink embedded in the polymer matrix. The electronic energy-level diagrams shown in Figure 5b are schematic representations illustrating the effective lowering of the electron energy level in the PVDF/SnO_2_ composite arising from interfacial charge redistribution. While direct molecular orbital calculations were not performed, the experimentally measured increase in work function (from 5.35 ± 0.02 eV to 6.37 ± 0.05 eV) provides strong evidence of modified electronic structure and enhanced electron-accepting capability in the composite. The observed work-function shift indicates a deeper electronic potential landscape at the surface, consistent with improved charge stabilization and enhanced triboelectric performance.

**Figure 5:**
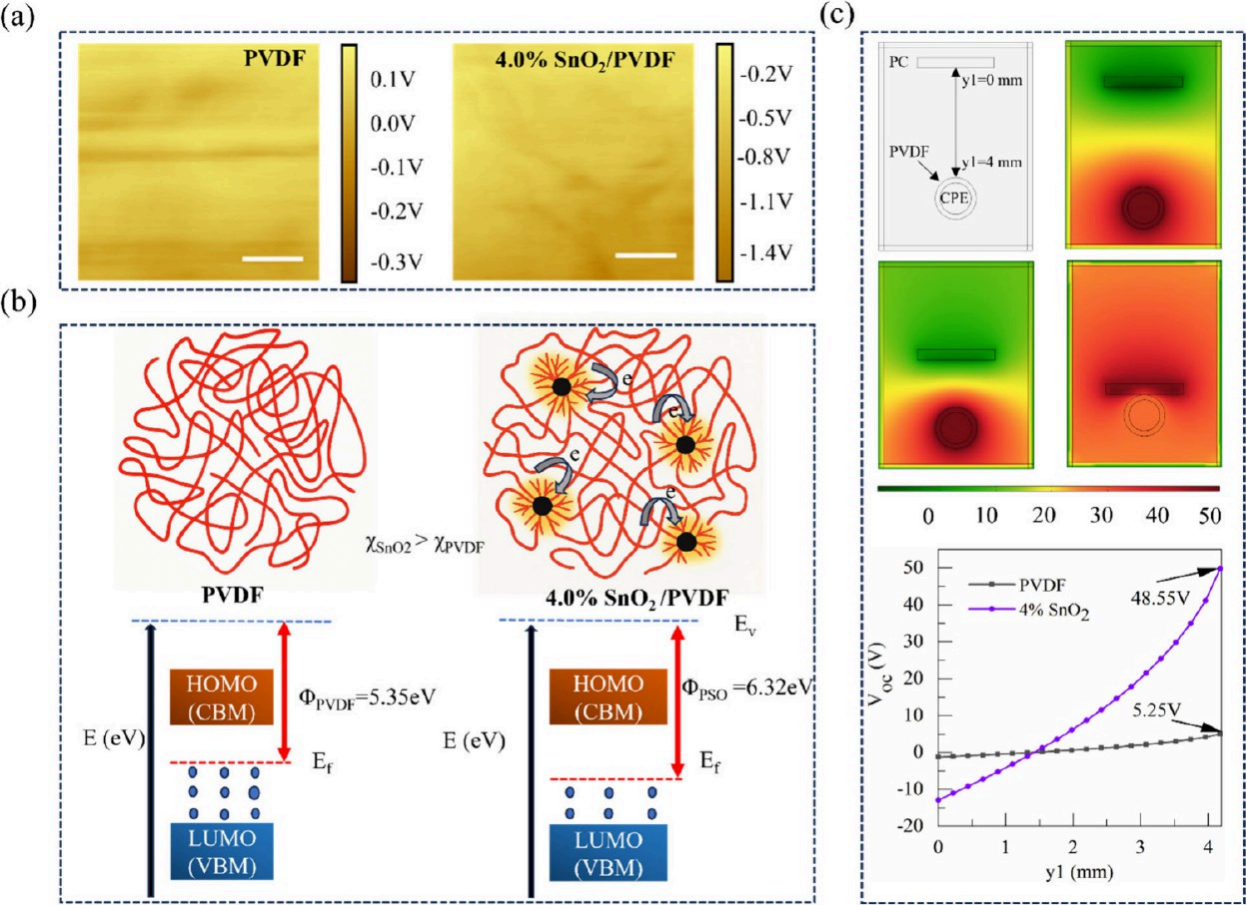
Comparison of experimental and numerical studies. (a) KPFM surface potential images of pristine PVDF and 4.0% PSO showing enhanced potential with SnO_2_ addition (scale bar: 1 μm). (b) Schematic illustration of PVDF and nanocomposite showing the interface interaction and change in work function due to nanofillers. (c) Simulated electric potential distribution and corresponding open-circuit voltage profile showing a significant enhancement (48.55 V) in 4.0% PSO compared to pristine PVDF (5.25 V).

Furthermore, using the experimentally determined dielectric constants and a parallel-plate approximation, the surface charge density σ can be estimated from Gauss’s law as

8 $$\sigma =\varepsilon_{0}\varepsilon_{r}E$$

where *ε*_0_ is the vacuum permittivity, *ε*_r_ is the relative dielectric constant of the film, and *E* is the average electric field across the film, and here it is equivalent to (CPD/*d*). The higher *ε*_r_ and larger surface potential shift of the PSO (2.55 mC/m^2^) composite translate into a significantly increased σ compared to that of pristine PVDF (0.15 mC/m^2^), confirming that SnO_2_ loading not only modifies the bulk dielectric response but also enhances interfacial charge accumulation and polarization, which are crucial for the improved electroactive and energy-harvesting performance of the nanocomposite.

Finite element analysis (FEA) was performed with COMSOL Multiphysics to reproduce the electrical response of the coaxial PVDF/cPE fiber under mechanical tapping. A two-dimensional axisymmetric model was constructed using the experimental geometry: an outer PVDF layer with a 0.9 mm radius and 50 mm length surrounding a cPE core. A PC plate was defined as a movable upper boundary to emulate the tapping motion applied in the real device. Material parameters were taken directly from experiments: a surface charge density from Table 1 and the dielectric constants from Figure 4. Figure 8c presents the calculated electric potential along the vertical axis (*y*_1_). The potential rises from approximately −8 V at the PC interface (*y*_1_ = 0 mm) to about 48.5 V at the fiber surface (*y*_1_ ≈ 4 mm), displaying a nearly exponential increase as the separation distance decreases for 4.0% PSO as compared to PVDF that gives voltage up to 5.25 V at contact. This strong gradient reflects enhanced charge induction as the PC plate approaches to the surface of either the pristine or nanocomposite PVDFs under open-circuit conditions. The spatial potential maps in Figure 5c illustrate the evolution of the electric field during successive PC positions. When the PC is far from the fiber, the potential is weak and largely confined near the coaxial core (green region). As the plate descends, the field intensifies, first appearing as yellow contours and then developing deep red zones around the fiber when contact is imminent, signifying a localized high potential. After contact and slight retraction, the field partially relaxes but remains elevated due to the retained surface charges. These simulations reproduce the magnitude and trend of the experimentally observed open-circuit voltage, confirming that the measured surface charge density and dielectric constant accurately describe the nanocomposite. The simulation also demonstrates an enhancement in the open-circuit voltage due to nanofiller reinforcement as shown plot of Figure 5c, which is in good agreement with the experimental results.

**Table 1:** Parameters of Calculation of Surface Charge Density and Work Function

parameters	Pt/Ir tip	PVDF	4.0% PSO
CPD	-	–0.15 ± 0.02 V	–1.12 ± 0.05 V
Φ_sample_	Φ_tip_ = 5.2 eV	5.35 ± 0.02 eV	6.32 ± 0.05 eV
d (tip–sample)	100 nm	100 nm	100 nm
σ	-	0.15 mC/m^2^	2.55 mC/m^2^

### 2.3 Electrical Characterization

#### 2.3.1 Dielectric Performance of TENG Fibers

The coaxial fiber architecture, comprising a central pristine PVDF or PSO dielectric core and an outer copper tape electrode, was developed to function analogously to a cylindrical or coaxial capacitor, as confirmed via impedance spectroscopy using an LCR meter (Figure 6a). Figure 6b shows that the equivalent circuit fitting parameters extracted from the Nyquist plots reveal systematic changes with increasing SnO_2_ incorporation. The fitting parameters are given in . The CPE magnitude associated with the primary dielectric response (*T*_CPE1_) increases progressively from 2.87 × 10^–12^ to 1.44 × 10^–11^ F·s^(p–1)^ as the SnO_2_ content increases from 0 to 4 wt %, indicating enhanced dielectric storage capability. This behavior is attributed to the increased interfacial polarization arising from the formation of microcapacitive interfaces between SnO_2_ nanoparticles and the PVDF matrix, consistent with Maxwell–Wagner–Sillars (MWS) polarization. The phase exponent values (*p* ≈ 0.96–0.99) remain close to unity across all compositions, suggesting that the dielectric response maintains quasi-ideal capacitive behavior despite nanoparticle incorporation. This indicates uniform dispersion without the formation of dominant conductive pathways. The bulk resistance (*R*) exhibits minor variation (50–55 Ω), while the high-resistance component (*R*_1_) shows fluctuations with filler loading, reflecting localized charge transport modulation and possible microstructural rearrangement at intermediate compositions. Notably, no dramatic reduction in resistance is observed even at 4 wt % SnO_2_, confirming that the system remains below the electrical percolation threshold and that dielectric enhancement dominates over conductive leakage. The secondary CPE term (*T*_CPE2_) also varies with SnO_2_ content, suggesting the modification of interfacial relaxation dynamics. Together, these parameter trends establish a direct correlation between nanoparticle-induced microstructural evolution and the enhanced dielectric behavior observed in PVDF/SnO_2_ nanocomposite fibers. Consistently, systematic incorporation of SnO_2_ nanofillers (0.5–4.0 wt %) results in a monotonic increase in capacitance and a corresponding decrease in impedance (Figure 6c), further supporting the role of interfacial polarization and space-charge accumulation under alternating electric fields. Together, these parameter trends establish a direct correlation between nanoparticle-induced microstructural evolution and the enhanced dielectric behavior of the PVDF/SnO_2_ nanocomposite fibers.^61,62^ While the nanocomposite remains below the percolation threshold, the slight decrease in bulk resistance with filler loading suggests improved local permittivity and a partial increase in leakage pathways. This dielectric enhancement has direct implications for the fiber’s performance as a TENG. In triboelectric systems, the output voltage and charge density are intimately linked to the dielectric constant and surface charge storage capacity of the active material. The elevated ε′ resulting from SnO_2_ incorporation increases the material’s ability to retain induced triboelectric charges and enhances the electrostatic field strength across the triboelectric interface. As a result, the nanocomposite fibers demonstrate a higher open-circuit voltage (*V*_oc_) and short-circuit current (*I*_sc_), thereby significantly improving TENG output without compromising mechanical flexibility or electrical insulation. The synergy between improved dielectric properties and triboelectric performance emphasizes the multifunctionality of SnO_2_-loaded PVDF fibers for energy harvesting applications in wearable electronics. The dielectric loss demonstrated by Figure 6d shows not much increment with the reinforcement amount of filler, and further the impedance shown in Figure 6e again shows the MWS effect as the impedance decreasing with the higher frequency.

**Figure 6:**
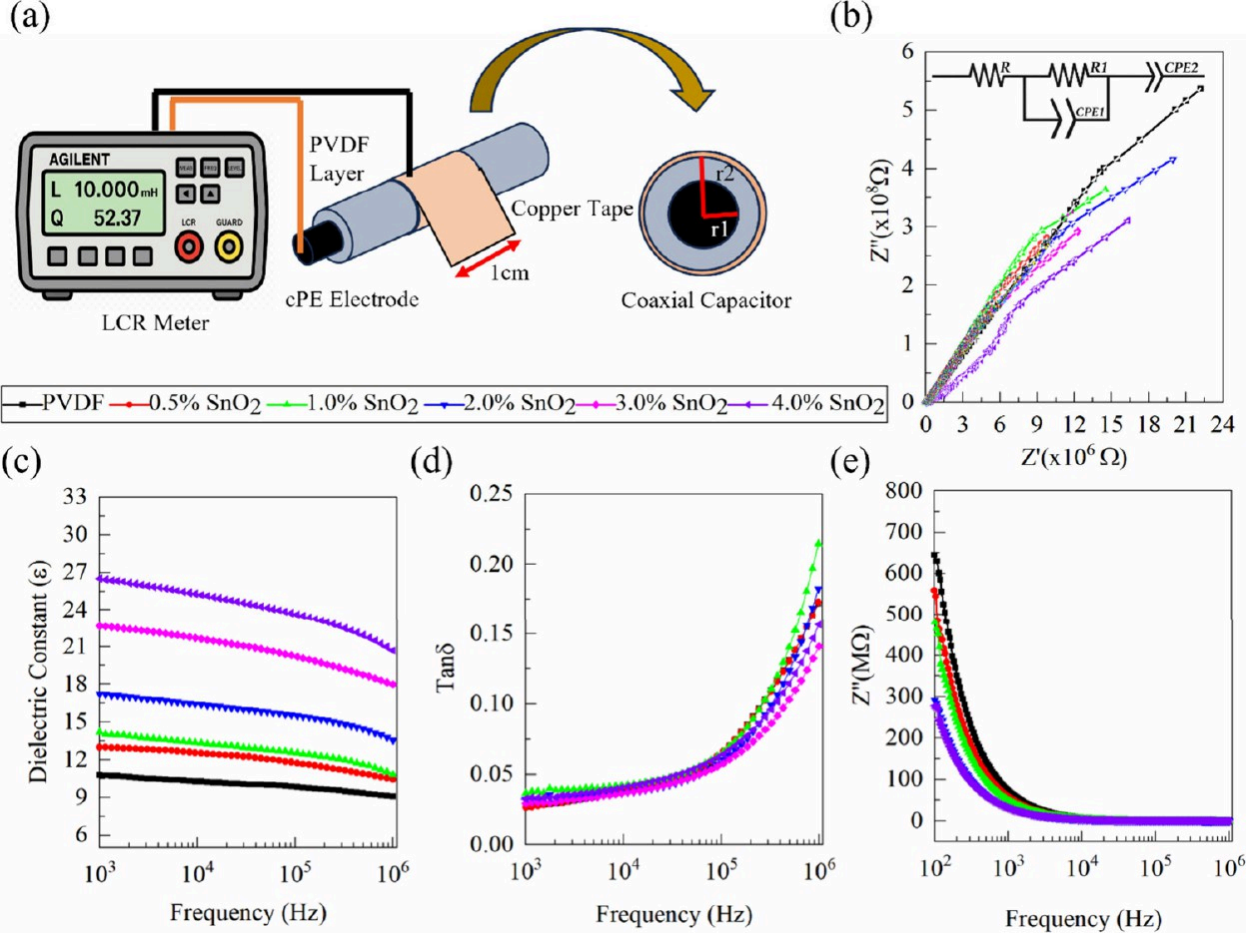
Dielectric properties of PSO fibers. (a) Schematic illustration of the coaxial capacitor device structure with PSO fibers and the measurement setup using an LCR meter. (b) Nyquist plots with equivalent circuit fitting for pristine PVDF and PVDF with varying SnO_2_ contents. (c) Frequency-dependent dielectric constant, (d) dielectric loss (tan δ), and (e) AC conductivity (*Z*′′) of the polymer nanocomposites.

#### 2.3.2 Electrical Performance of TENG Fibers

The electrical performance of a single fiber was evaluated by measuring the open-circuit voltage, short-circuit current, and power density. The fabricated pristine PVDF or PSO TENG fiber functions through a single-electrode (SE) mode, where output generation arises from the combined effects of contact electrification and electrostatic induction. In this configuration, one triboelectric layer makes direct contact with the electrode, while the other layer remains isolated. The repeated contact and separation of the layers create an electrostatic imbalance, which drives charge transfer between the electrode and the ground. In the initial state (noncontact), the system is in electrostatic equilibrium, meaning no charge exchange occurs between PVDF or PSO and PC, and no potential difference exists between the electrode and the ground (Figure 5a). When an external mechanical force brings PSO into contact with PC, charge transfer takes place due to the differing triboelectric polarities of the two materials. Electrons transfer from PC to PVDF or PSO, resulting in a net negative charge on PVDF or PSO and a corresponding net positive charge on PC. At this compressed stage, the opposite charges are confined to the surfaces of the materials, creating a net zero potential difference. Thus, the system remains balanced (Figure 7a). Since both PVDF or PSO and PC are insulating materials, they can retain charges for extended periods. However, when separation between the two layers begins, *V*_oc_ increases until it reaches its maximum value. This separation disrupts the electrostatic balance and induces a potential difference, causing electrons to flow from the electrode to the ground through electrostatic induction. Once the electrons flow out, electrostatic equilibrium is re-established, and no further electron movement occurs at this stage. As the PVDF or PSO layer starts moving back toward the PC surface, the system’s equilibrium is again disrupted because the electrode now has a lower electrical potential. This triggers electron movement from the ground back to the electrode, generating an instantaneous current in the opposite direction. When the two layers come into contact again, electrostatic equilibrium is restored. The alternating movement of electrons in opposite directions during the contact-separation cycle produces an AC signal.

**Figure 7:**
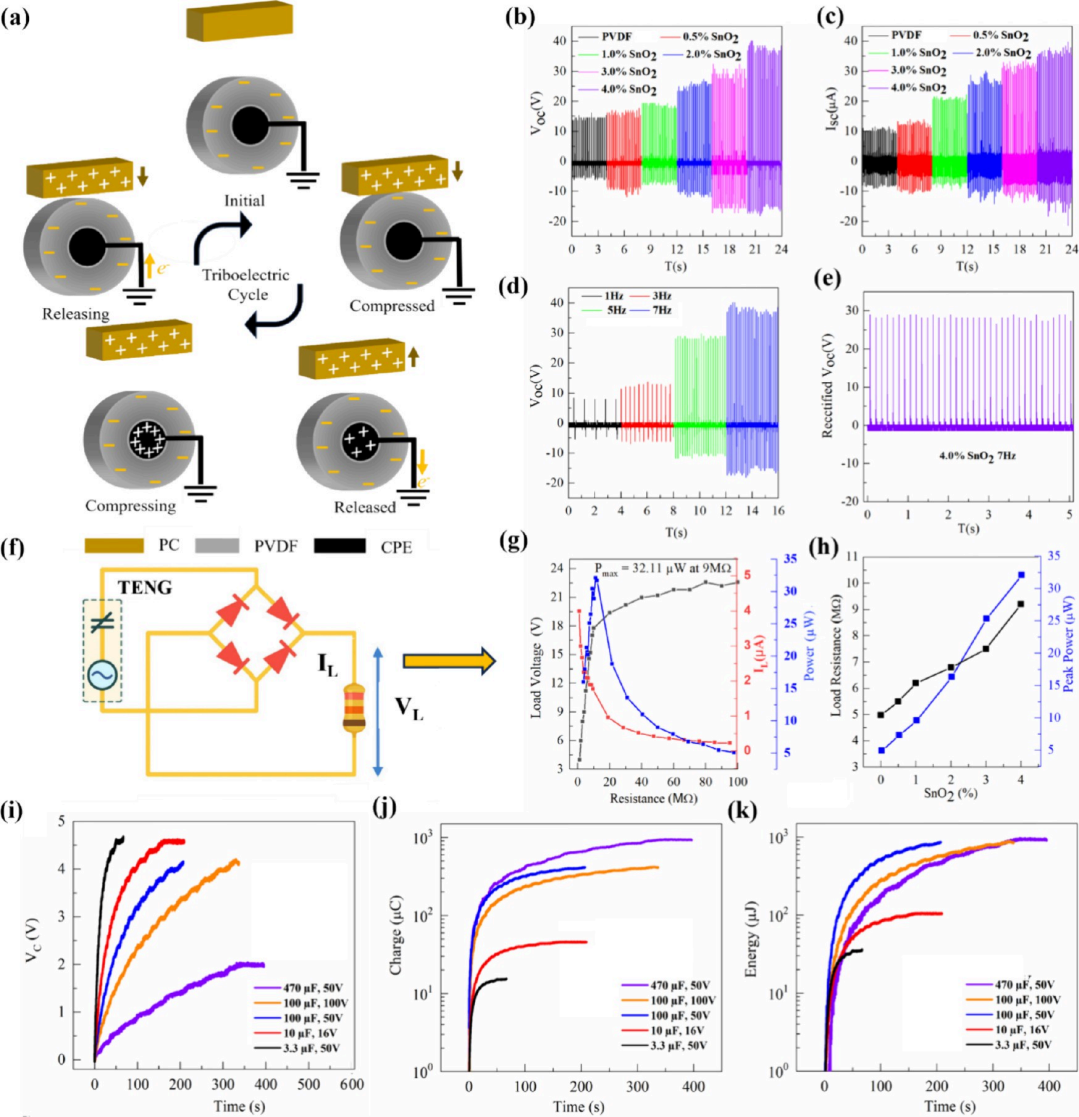
Electrical characterization of TENG fibers. (a) Schematic illustration of single-electrode mode TENG fiber. (b) The open circuit voltage on the 7 Hz frequency for all the composition. (c) The short circuit current on the 7 Hz frequency for all compositions. (d) The open circuit voltage 4.0% PSO. (e) Rectified open circuit voltage 4.0% PSO at 7 Hz. (f) Circuit used to measure power. (g) The load voltage, load current, and peak power at matched impedance, (h) the power measurement for the 4.0% PSO with load resistance, (i) voltage–time (*V*–*t*) charging curves across the capacitor, (j) charge–time curves across the capacitor, and (k) energy–time curves of different capacitors charged by the TENG fiber.

Figure 7b presents the peak-to-peak voltage (*V*_pp_) responses of pristine PVDF and PSO nanocomposite fibers at various frequencies (7 Hz). For pristine PVDF, the *V*_max_ values increase from 10.4 V at 7 Hz. However, with the addition of SnO_2_ nanofillers at 4.0%, the *V*_max_ significantly increases, reaching 8.31 V at 1 Hz and peaking at 37.2 V at 7 Hz as shown in Figure 7c and d. This enhancement demonstrates the superior triboelectric performance of PSO composites fiber compared to pristine PVDF fiber. The voltage output also exhibits a frequency-dependent trend, with higher frequencies generating stronger responses. The results highlight the role of SnO_2_ nanofillers in improving the polarization and triboelectric properties of the PVDF matrix, particularly at higher concentration. *I*_sc_ for the polymer nanocomposites increases significantly with the addition of SnO_2_ nanofillers into the PVDF matrix, as shown in Figure 7c. For pristine PVDF, the maximum *I*_sc_ values at 7 Hz is relatively low, with the highest recorded at 10.11 μA. Upon incorporating SnO_2_ at 0.5%, there is a noticeable increase in the *I*_sc_, indicating enhanced triboelectric performance. As the SnO_2_ content increases to 1.0, 2.0, 3.0 and 4.0%, the I_sc_ values further improve, reaching a maximum of 36.25 μA at 7 Hz for 4.0% PSO. This enhancement can be attributed to the improved dielectric, charge density and polar properties of the nanocomposite, which facilitate greater charge generation and transfer under mechanical excitation. Further, electrical performance stability was measured using both the tapping device at 5 and 10 Hz frequency by running them for 25 min as shown in . The mechanical stability and bending test were done using the stepper motor, and the fiber was arranged in such a position that we can give repetition bending to the fiber as given in and also in .

The performance of a TENG is therefore strongly influenced by the capacitive behavior, dielectric constant, and surface charge density of the functional surface (). In pristine PVDF, polarization arises mainly from electron displacement and the dipole orientation. In contrast, the PSO nanocomposite introduces an additional contribution from interfacial polarization, which further enhances both the dielectric constant and the charge density. The uniform distribution of SnO_2_ nanofillers within the PVDF matrix leads to the formation of microcapacitor-like structures, where electroactive β-phase PVDF regions are sandwiched between adjacent SnO_2_ layers, resembling parallel-plate capacitors. These microcapacitors act as charge-trapping sites, thereby increasing both capacitance and surface charge density. The surface charge density in a TENG is determined by the interplay between the charge generation and charge decay. Charge decay occurs through three main mechanisms: (i) air breakdown, in which accumulated surface charges are neutralized through ionization of nitrogen molecules in air; (ii) charge drift, where surface charges migrate along the electric field between oppositely charged surfaces, leading to partial neutralization; and (iii) charge diffusion, which results from a concentration gradient, allowing electrons to diffuse into the bottom electrode.^45^ A stable electrical output is achieved when a dynamic equilibrium is established between charge generation and these loss pathways. In addition to these effects, the electronic structure of the nanocomposite influences the triboelectric output. A reduction in the lowest unoccupied molecular orbital (LUMO) energy level increases the electron-accepting capability, enabling electrons to occupy a lower energy state and thus enhancing the charge stabilization. SnO_2_ and related nanostructures are known to lower the LUMO energy level, which contributes to improved charge-trapping efficiency in the PSO system.^65^

The performance of PSO fibers is also sensitive to the operating frequency. At higher contact–separation frequencies, the reduced charge decay time results in greater triboelectric charge retention, thereby improving the charge density. Conversely, at lower frequencies, the charge leakage during each cycle diminishes the overall output. The rectified output signals are presented in Figure 7e, where the generated voltage is shown as a positive waveform. The corresponding circuit diagram of the fiber connected to a rectifier and load resistance is provided in Figure 7f, which was used to evaluate the power delivery. The maximum power output occurs when the impedance of the fiber matches the external load resistance. As shown in Figure 7g, the 4.0% PSO fiber produced a peak power of 32.11 μW at 9 MΩ under a tapping frequency of 7 Hz. The trend in Figure 7h further illustrates the progressive enhancement in peak power output as the SnO_2_ reinforcement increases from pristine PVDF to 4.0% PSO. This increase in power output is accompanied by a rise in impedance, reflecting the capacitive nature of the polymer nanocomposite. Furthermore, to demonstrate the energy harvesting and applicability, 18 LEDs were used in 3 lines parallel with 6 LEDs in series as shown in and connected to the circuit shown in Figure 7f or . The LEDs were blinking as the rectified signal was spiked; however a polypropylene film capacitor was connected to the given circuit, and the signal was a proper DC as shown in , resulting in continuously lighting the LEDs. Moreover, the setup was demonstrated in .

To further analyze the charge-generation behavior of the fiber, different capacitors were charged using the TENG fiber with the highest performance, evident from Figure 7i. The obtained voltage–time curves (*V*–*t* curves) of charging of various capacitors provide critical insights into the dynamic behavior of the energy transfer process shown in Figure 7j. Each *V*(*t*) curve reveals distinct charging characteristics governed by the interplay between the TENG’s output and the load capacitance. The initial slope d*V*/d*t* on the *V*(*t*) curve reflects the rate at which charge is delivered to the capacitor, effectively capturing the fiber’s instantaneous charge generation capability. It has been observed that the smaller capacitors exhibit steeper initial slopes and reach higher voltages rapidly, whereas larger capacitors display a slower rise in voltage, indicative of their higher energy storage capacity with higher impedance but lower voltage gain under the same TENG excitation. The nonlinear shape of all curves deviating from the classical exponential trend expected in RC charging circuits suggests a nonconstant current and power output, characteristic of the pulsed, high-impedance nature of TENG sources. To further quantify the TENG performance, the effective current *I*(*t*) at any time point can be approximated by

9 $$I\left(t\right)=C\cdot \frac{dV}{dt}$$

using the instantaneous slope of the *V*(*t*) curve and the known capacitance. This provides a time-resolved measure of the TENG’s ability to inject charge into each capacitor, as shown in Figure 7k. Furthermore, the stored energy within the capacitor at any time is given by

10 $$E\left(t\right)=\frac{1}{2}CV{\left(t\right)}^{2}$$

To get energy (*E*(*t*)) vs time plots that reveal the rate of energy accumulation and power delivery efficiency of TENG fiber, Figure 7j and k have logarithmic *Y* axis to differentiate the curves’ position. The comparative analysis of energy profiles across capacitors of varying sizes emphasizes a key design insight: while smaller capacitors reach higher voltages quickly, larger capacitors, despite slower charging, ultimately store greater energy. This behavior implies that the TENG functions more effectively as a high-voltage, low-current energy source, favoring load configurations that optimize both voltage rise and energy capacity.

The open-circuit voltage of the nanocomposite fiber by FEA simulation demonstrated in Figure 5c is somewhat higher as compared to that experimentally, indicating that environmental and other factors may have influenced the experimental measurements; the simulation shows the maximum possibility of open circuit voltage generation with the same area and other surface conditions while in contact separation mode. The results verify that the coaxial PVDF/cPE architecture concentrates the electric field near the core and efficiently converts the mechanical tapping into a high electrical potential, demonstrating the suitability of the design for tactile sensing and triboelectric energy-harvesting applications.

### 2.4 Sensitivity of TENG Fibers

To obtain a realistic evaluation of the TENG fiber’s sensitivity under low-force and ambient conditions, we performed two distinct measurements using a lightweight Mylar balloon. The first involved a conventional contact–separation mode, while the second simulated gesture-based sensing by allowing the balloon to swing freely over the PSO fiber without making physical contact. Notably, the balloon material Mylar, a composite of nylon with polychloroprene or 2-methyl-1,3-butadiene, is not considered highly positive in the triboelectric series, making it a conservative test counter-material. As shown in Figure 8a and b, the TENG fiber demonstrated a clear voltage output in both configurations. In the contact–separation mode, pronounced and repeatable voltage signals confirmed strong triboelectric activity. In contrast, during the swinging-over mode, the absence of direct contact led to smoother signal profiles, likely due to air dielectric breakdown and reduced field confinement, which suppressed sharp peak formation. Despite this, measurable signal amplitudes approaching ∼1 V were consistently observed, emphasizing the high intrinsic sensitivity of the TENG fiber even under soft or noncontact mechanical stimuli. These results highlight the fiber’s potential for use in ambient, wearable, or gesture-responsive applications, where minimal physical interaction is desired or unavoidable. The balloons’ contact separation mode and swinging over mode can be seen in .

**Figure 8:**
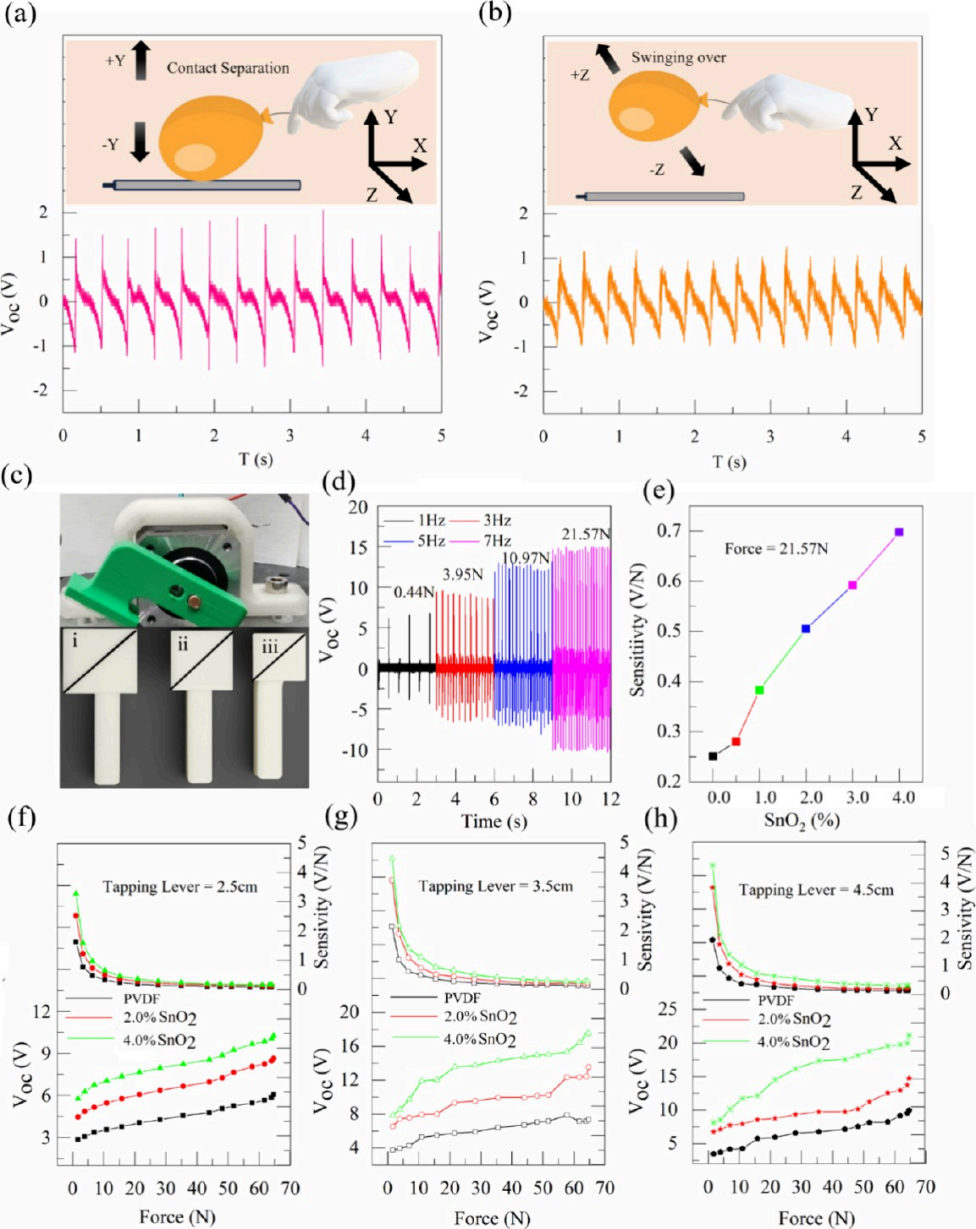
Electrical characterization of fibers. (a) Output voltage of the TENG fiber under contact–separation mode along the *Y*-axis with the balloon. (b) Output voltage under swinging-over mode along the *Z*-axis. (c) Experimental setup with inclined surfaces for force application (lever diagonal length *i* = 2.5 cm. *ii* = 3.5 cm, *iii* = 4.5 cm). (d) Output voltage at different applied forces and frequencies. (e) Sensitivity variation with the SnO_2_ reinforcement content. Voltage–force and corresponding sensitivity curves for pristine PVDF, 2.0% PSO, and 4.0% PSO nanocomposite fibers with (f) 2.5 cm, (g) 3.5 cm, and (h) 4.5 cm tapping levers.

The triboelectric response of PSO fibers under mechanical force has been characterized in terms of voltage and current output. TENG systems generally generate large values of open circuit voltage compared to the short circuit current. This is due to the capacitive and electrostatic behavior of the TENG during the contact separation process. The conventional solids have a surface with limited roughness filled with air, causing charge breakdown and leading to hindrance of the charge transfer. To overcome this, large mechanical loads are generally applied to push the material to generate higher voltages. However, within the demonstrated setup, the output response does not increase proportionally with a higher applied load. While the voltage continues to rise with increasing force, a noticeable change in trend occurs beyond a certain level, where the rate of increase becomes significantly slower, even though the material, contact area, and surface roughness remain constant.

Several studies have investigated the relationship between the applied mechanical load and output voltage in TENG systems. However, to the best of our knowledge, no prior work has formally reported or systematically demonstrated this behavior through controlled experimental protocols. As a result, there remains no widely accepted framework or standardized expression relating applied force voltage-per-newton sensitivity in TENG devices. Therefore, in this study, a systematic experimental approach was undertaken to establish a quantitative relationship between the applied force and the resulting voltage sensitivity, defined as the ratio of the open-circuit voltage to the applied mechanical force (*V*/*N*). Experimental measurements were conducted by applying calibrated tapping forces using a stepper motor controlled by the Arduino microcontroller with varying frequency from 1 to 20 Hz in the range of 0.4 to 62 N force on the TENG fibers. The 3D printed levers with three different dimensions as labeled with the diagonal length (2.5 cm, 3.5 and 4.5 cm long black lines in Figure 8c) were used to apply the tapping on the fiber. Figure 8d displays the measured voltage output as a function of the applied force. Using these measurements, the sensitivity was calculated for different filler contents and is presented in Figure 8e. The trend demonstrates that the sensitivity improves with increasing reinforcement in the polymer matrix. Further, the corresponding sensitivities were extracted from the voltage output per unit force as shown in Figure 8f–h, yielding the following empirical data set. The resulting trend exhibits a nonlinear decay in sensitivity with increasing applied force, suggesting a sublinear electromechanical coupling behavior. To describe this relationship quantitatively, a power-law model was fitted to the data by

11 $$S\left(F\right)=a\cdot F^{-b}$$

where *S*(*F*) is the sensitivity (*V*/*N*), *F* is the applied force, and *a* and *b* are empirical fitting parameters. A least-squares fit yielded for the 4.0 wt % PSO by

12 $$S\left(F\right)=7.17\cdot F^{-0.78}$$

This expression captures the essential physics of the polymer-based triboelectric response: as the applied force increases, the effective sensitivity decreases due to saturation of surface charge generation and electrostatic potential, possibly coupled with changes in the contact area and material stiffness. The exponent *b* ≈ 0.78 highlights the nonlinear but monotonic decay, characteristic of soft, elastomer-based triboelectric systems. The parameter *a* represents the sensitivity scale at the initial force or low forces and is governed by material tribopolarity, surface roughness, elasticity, dielectric properties, and fiber geometry. A higher *a* indicates a greater voltage output per unit force in the low-force regime, crucial for soft or ambient sensing. The exponent *b* characterizes the rate of sensitivity decay with increasing force, reflecting nonlinear electromechanical coupling due to surface charge saturation, limited contact area growth, and elastic limits. A larger *b* denotes a faster sensitivity reduction under higher loads. To verify this phenomenon further, the previously demonstrated work has been chosen, and their data are replotted as given in Figure 9. Force–sensitivity characterization of thermally drawn TENG fibers fitted with the empirical power-law model is given in eq 11. The high fitting accuracy confirms the validity of this empirical force-sensitivity law, as shown in Figure 9.

**Figure 9:**
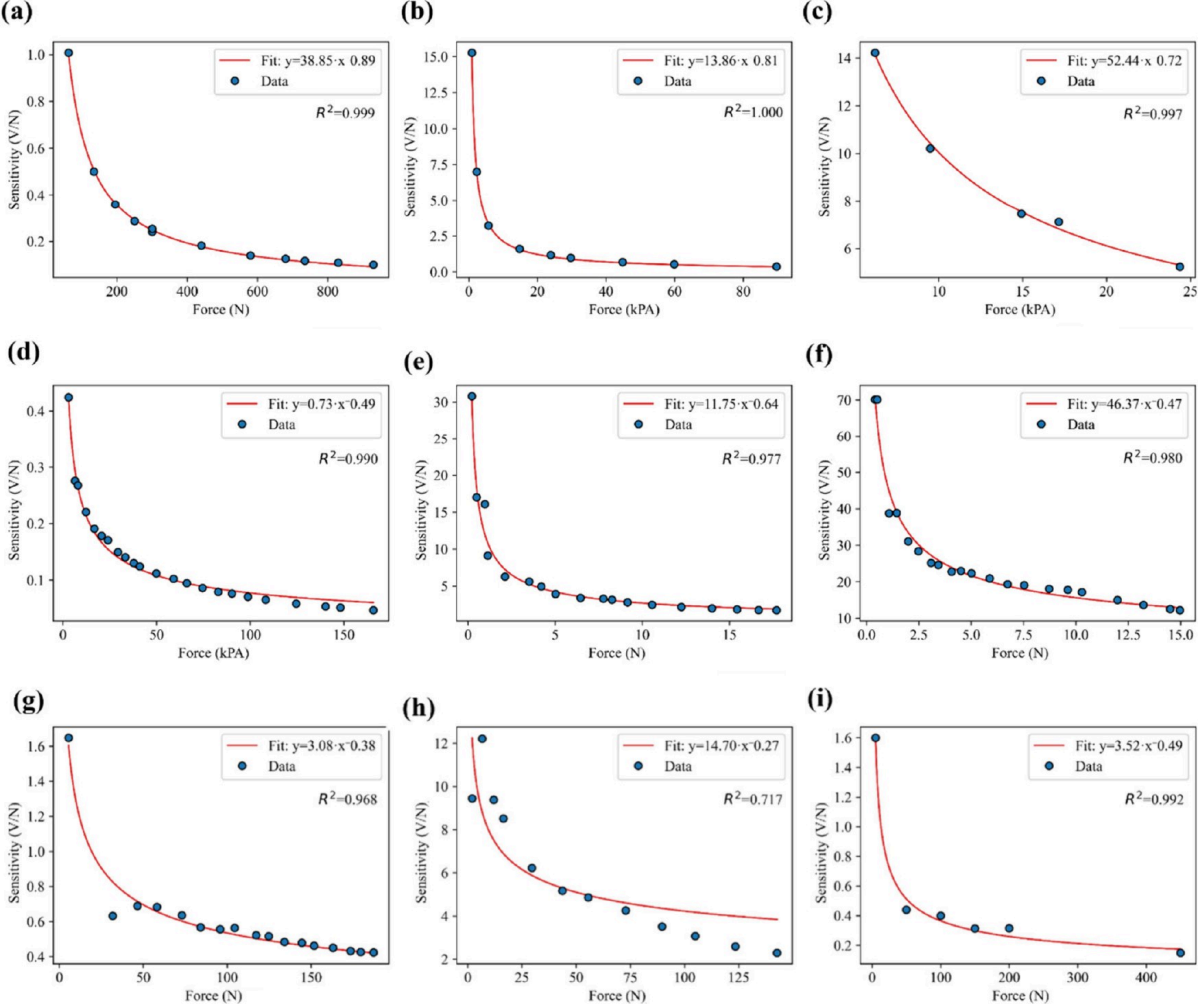
Fitting the power law in sensitivity vs force curve for (a) TENG based on Cu and polyethylene terephthalate (PET),^66^ (b) silver nanowires (AgNW)/styrene ethylene butylene styrene (SEBS) layer encapsulated in polydimethylsiloxane (PDMS),^67^ (c) ionogel (IG)-TENG made of 1-butyl-3-methylimidazolium bis­(trifluoromethylsulfonyl) imide (BMIM Tf_2_N) ionogel between Ecoflex layers with a Cu electrode,^68^ (d) AgNW-PDMS upper layer with Cu film and PDMS lower layer with patterned AgNW electrodes,^69^ (e) silica gel–copper based TENG,^70^ (f) Ag-textile and PDMS-based TENG,^71^ (g) PDMS replicated from silicon template,^72^ (h) PET–Ag/indium tin oxide (ITO)–PDMS based TENG,^73^ (i) PDMS-carbon nanotubes (CNT) based TENG.^74^

Together, these parameters describe the fundamental behavior of the TENG and guide the design of responsive, low-force sensing systems. Figure 10a–i shows the sensitivity of our PVDF and its nanocomposite fibers. Figure 10j–l shows the comparison of previously demonstrated TENG studies, indicating strong and consistent nonlinear behavior. The sublinear exponent confirms stable polarization saturation with increasing force, demonstrating efficient electromechanical coupling and mechanical compliance of the PVDF fiber. To our knowledge, this is the first empirical formulation of a force–sensitivity law specifically tailored for thermally drawn triboelectric fibers. The law is supported by excellent fitting accuracy, and its functional form is consistent with previously observed behavior in surface contact-mode TENGs, where the electrostatic output saturates with increasing mechanical input due to limited tribo-surface charge density and elastic recovery constraints. This power-law relationship not only provides a standardized metric for sensitivity characterization but also opens the way for quantitative design rules in sensor applications involving soft TENG fibers. It can serve as a foundational model for evaluating the performance of future TENG-based tactile or pressure sensors in robotics, prosthetics, and biomedical systems.

**Figure 10:**
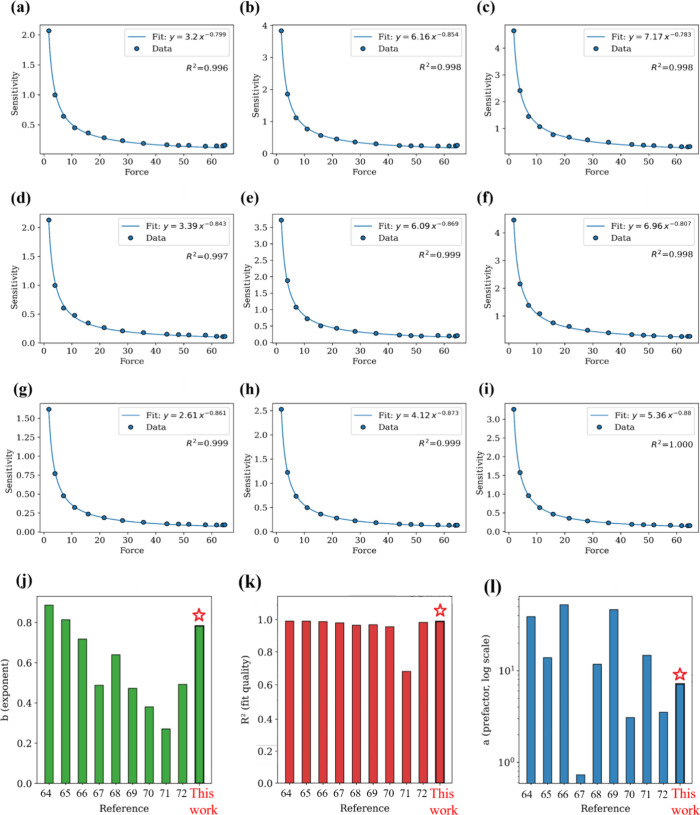
Power-law fitting of the sensitivity (*V*/*N*) vs force (*N*) curves for PVDF–SnO_2_ fibers: PSO 4.0% SnO_2_ with (a) 2.5 cm lever, (b) 3.5 cm lever, and (c) 4.5 cm lever; PSO 2.0% SnO_2_ with (d) 2.5 cm lever, (e) 3.5 cm lever, and (f) 4.5 cm lever; and pristine PVDF with (g) 2.5 cm lever, (h) 3.5 cm lever, and (i) 4.5 cm lever. (j) Comparison of the *b* component of the power law, (k) comparison of *R* ^2^ (fit quality), and (l) comparison of the parameter (on a logarithmic scale).

### 2.5 Application of TENG Fibers in Continuum Robot

The high β-phase content and enhanced dielectric constant obtained for the PSO composite translate directly into a practical sensing capability when the fiber is routed as a triboelectric wire sensor (TWS) along continuum robots. The fibers were mechanically laid by threading three TWS channels through special through-holes on each disc of the robot’s support structure and wrapped around the disc in the manipulator’s support structure. This method physically secured the fibers to the robot structure, thereby reducing the likelihood of motion artifacts or noise that could arise from loose wiring. To elucidate the integration strategy, Figure 11a presents the overall CAD view of the smart in-pipe inspection robot, highlighting the integration of the continuum manipulator within the tracked robotic platform. The continuum manipulator, constructed using a cable-driven, flexible backbone, serves as the central mechanism for navigating bends, constrictions, and complex geometries inside pipes. This figure illustrates how thermally drawn PSO triboelectric wire sensors (TWS) are embedded along the manipulator to enable self-powered tactile perception during in-pipe locomotion. The robot’s outer tracked system provides propulsion, while the embedded continuum arm performs inspection, obstacle avoidance, and contact detection tasks. Figure 11b shows the structural layout of the continuum manipulator, specifically the trimmed-helicoid skeleton through which the TENG fibers are routed. The manipulator consists of repeating disk segments connected by a helical backbone, enabling smooth multi-DOF bending. The designated TENG wire attachment zones are highlighted, indicating where the PSO fibers are mechanically fixed along the outer surface. These attachment sections ensure consistent contact with the environment and allow the TENG wires to generate tactile signals during motion. The figure also marks the first section and the end-effector region, clarifying how the sensor routing aligns with the mechanical structure of the robot. The configuration of the TWS channels is such that three channels are threaded through dedicated through-holes on each disk (Figure 11c) and terminated on an Arduino Mega 2560 via 3 MΩ load resistors (Figure 11d). It has been demonstrated that under the condition of periodic tapping (2 Hz), each channel delivers clean, broadband voltage peaks (>8 V) that rise well above the baseline noise (σ ≈ 0.18 V). This phenomenon is illustrated in Figure 11e.

**Figure 11:**
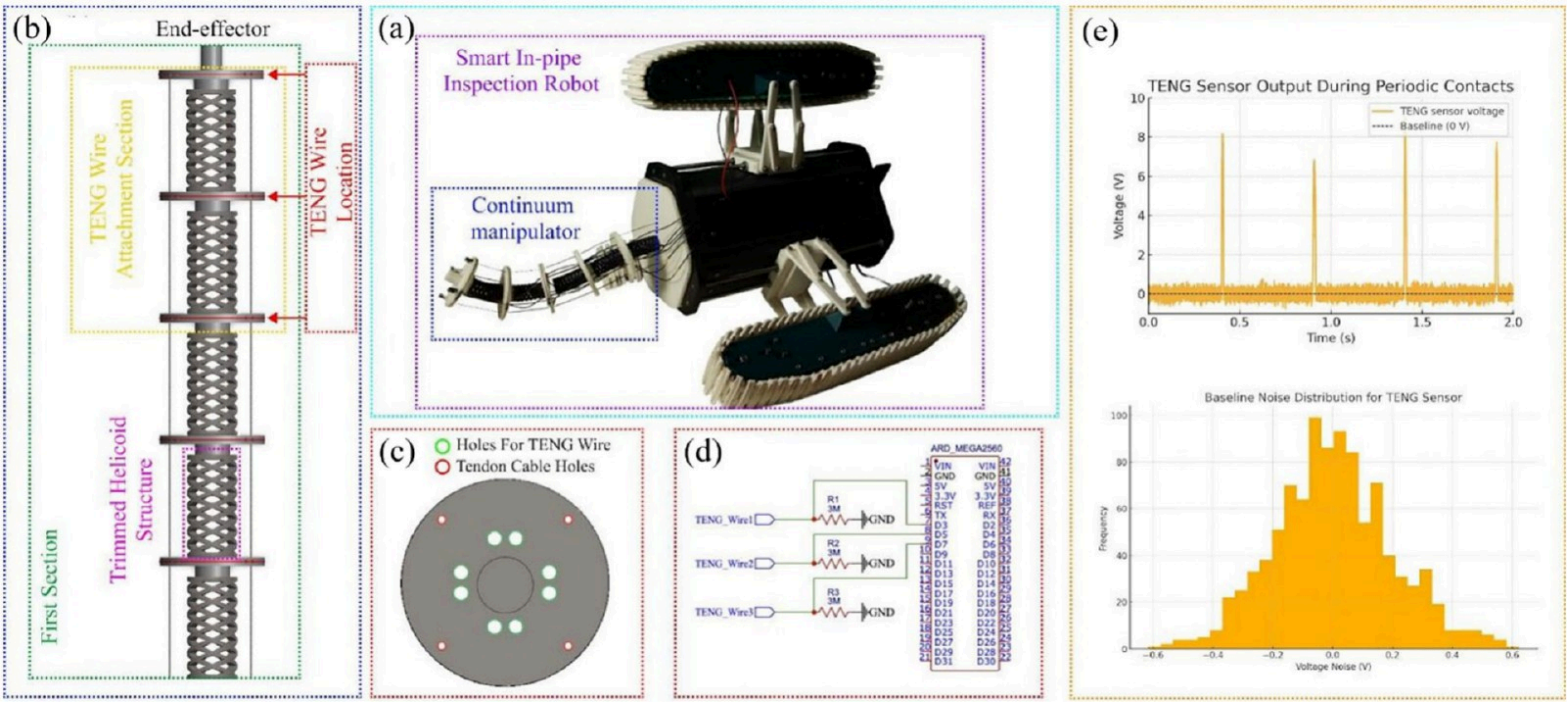
Integration of PVDF-SnO_2_ TENG wire sensors into the smart in-pipe inspection robot: (a) CAD rendering of the robot with continuum manipulator; (b) trimmed-helicoid backbone showing TENG-attachment zones; (c) disk design with through-holes for TENG routing (green) and tendon cables (red); (d) three-channel wiring diagram (3 MΩ load resistors to Arduino Mega 2560); (e) representative open-circuit voltage waveform during periodic contacts and baseline-noise histogram.

Further, Figure 12 provides a simplified schematic of the TWS operation, where the triboelectric fiber channels detect contact-induced voltage spikes (Figure 12a), which are processed through the microprocessor’s analog and digital pathways (Figure 12b) to trigger immediate actuation shutdown when external contact occurs (Figure 12c). This illustration clarifies the complete sensing-to-stop workflow of the robot and highlights how the fiber serves simultaneously as a tactile sensor and a self-powered safety mechanism for continuum robots.

**Figure 12:**
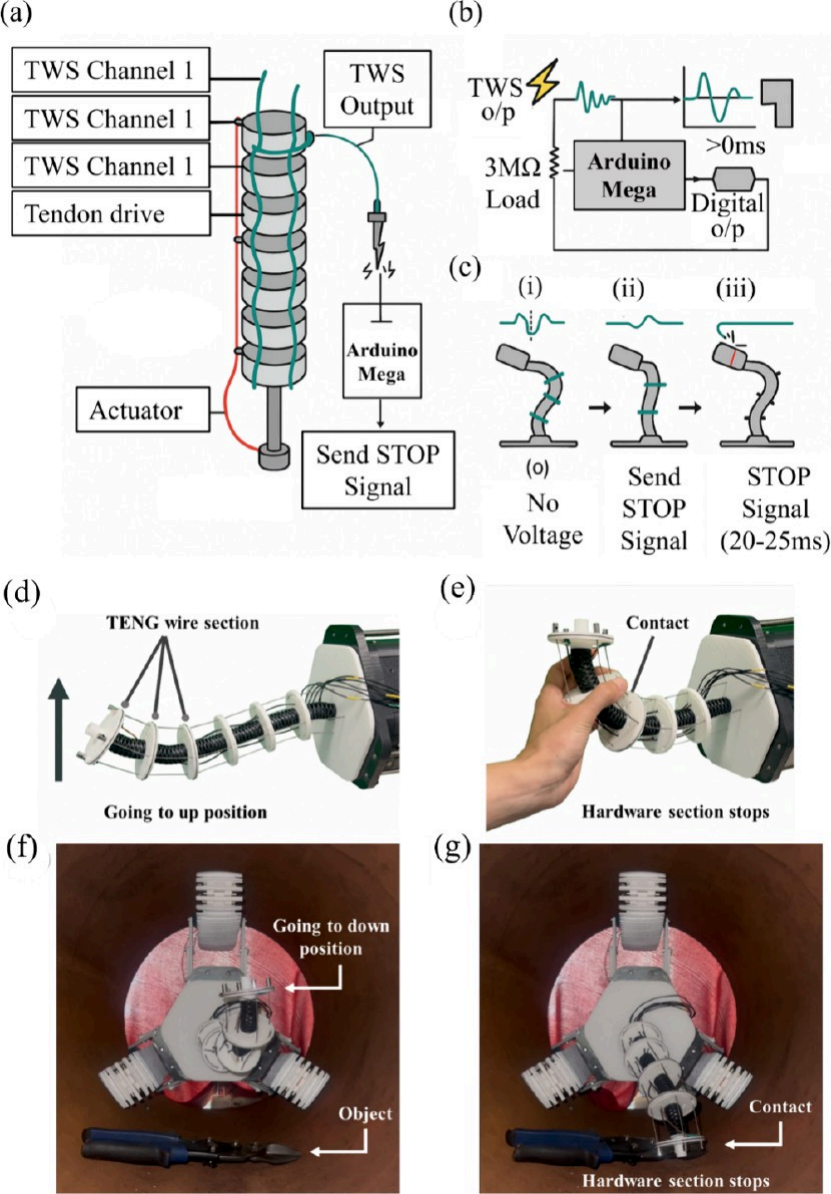
Schematic of the TWS operation for the continuum robot. (a) The robot equipped with TWS channels generates triboelectric voltage spikes upon external contact. (b) These signals are conditioned and interpreted by the Arduino-based electronics, which monitor threshold-based events in real time. (c) Contact-induced voltage peaks trigger an immediate STOP command to the actuator, enabling self-powered, rapid collision detection. (d) Self-powered collision detection of the continuum manipulator in free space: (d) upward motion with no contact; (e) contact with a human hand induces a voltage spike that triggers an immediate hardware stop. In-pipe collision-detection scenario: (f) the manipulator bypasses a loose tool without contact and (g) contact between the tool and the TENG wire sensor.

As illustrated in Figure 12d–g, a free-space experiment was conducted in which the TWS was spirally wrapped through the trimmed-helicoid backbone of a cable-driven continuum manipulator (with an outer diameter of 25 mm). In the absence of contact (Figure 12d), the manipulator moves in an upward direction at a rate of 8 mm s^–1^. However, when a human hand encountered any exposed segment of the fiber, a voltage spike of approximately 7 V (peak-to-baseline) was generated, and the microcontroller issued an immediate hardware stop signal (Figure 12e). The mean response latency, measured from first contact to motor shutdown, was 24 ± 3 ms. As demonstrated in Figure 12f, this concept is replicated within a 140 mm-ID steel pipe, which is the target scenario for the development of the smart in-pipe inspection robot, particularly in confined environments with conductive surfaces, which pose significant challenges for conventional rigid electronic systems. Initially, a stray tool positioned on the pipe floor is bypassed safely (Figure 12g). Subsequent to the aforementioned collision, the TWS registers a 6–9 V transient and halts the advance within one control cycle (20 ms), thus preventing mechanical jamming (Figure 10d). The outcomes of this study demonstrate that the mechanically robust, centimeter-scale PSO fibers fabricated in this research are well-suited for distributed, self-powered collision detection in soft and continuum robotic systems. In comparison with conventional strain gauges or flexible piezoresistive films, the proposed TWS does not require external power or additional signal-conditioning ICs, thereby reducing wiring complexity and enhancing intrinsic safety in confined, conductive environments, such as metallic pipelines.

## 3 Conclusion

This study demonstrates a new benchmark in sustainable TENG fiber fabrication by reinforcing green-synthesized SnO_2_ nanofillers into PVDF via scalable thermal drawing. The optimized 4.0% PSO fibers exhibited an open-circuit voltage of 37.2 V, a short-circuit current of 36.25 μA, and a peak power density of 32.11 μW at 9 MΩ, representing more than a 3.5-fold increase in power output compared to pristine PVDF fibers. The β-phase content increased from 29.6% (film) to 46.2% (fiber), while dielectric constant values rose by 3 times, directly enhancing charge storage and transfer. Importantly, the fitting of a force–sensitivity power law provides the first standardized metric for electromechanical coupling in thermally drawn TENG fibers, enabling predictive sensor design. Qualitatively, this work demonstrates the first evidence of spherulitic microstructure formation under a thermal drawing process, revealing how nanofiller–polymer interactions and extensional flow synergistically drive β-phase crystallization. The ecofriendly synthesis route emphasizes sustainability without compromising performance. Application studies in continuum robots further validated the fibers’ robustness, with collision detection achieved in <25 ms and reliable operation in both free-space and confined pipe environments. Overall, this work not only surpasses state-of-the-art PVDF-based TENG fibers in both energy output and sensing precision but also introduces a scalable, green, and multifunctional platform for self-powered robotics, biomedical devices, and wearable electronics.

## 4 Experimental Section

### 4.1 Materials

Polyvinylidene fluoride (PVDF) and polycarbonate (PC) were obtained from Ajedium Films, and dimethylformamide (DMF) was obtained from Carlo Erba Reagents S.A.S. Tin­(II) chloride dihydrate (SnCl_2_·2H_2_O) with 99.99% purity was purchased from Sigma-Aldrich, and DCM was obtained from Merck KGaA. Bananas were procured from a local market. All chemicals were used without further purification, while banana peels were thoroughly washed with deionized (DI) water prior to use.

### 4.2 Synthesis of SnO_2_ Nanopowders

The SnO_2_ were synthesized following a previously reported green route using banana peel extract as a biological reducing and stabilizing agent,^75^ with slight modifications. Briefly, the extract was prepared by placing 5 g of banana peel powder in 50 mL of distilled water and heating the mixture at 100 °C for 3 h. The obtained suspension was filtered, and the clear filtrate was concentrated using rotary evaporation to yield a yellow powder, which served as the bioactive reducing agent for SnO_2_ formation. For the synthesis, 1 g of SnCl_2_·2H_2_O was dissolved in 10 mL of distilled water, followed by the addition of 1 g of the yellow extract powder under continuous stirring. The mixture was maintained at 80 °C for 3 h to promote the formation of SnO_2_ nanoparticles. The resulting product was centrifuged at 6000 rpm for 10 min, washed several times with distilled water to remove residual impurities, and finally dried in an oven at 150 °C overnight to obtain the SnO_2_ nanopowder.

### 4.3 Fabrication of Polymer/SnO_2_ Nanocomposite Fibers

The fabrication of the sensor begins with preparing the nanocomposite film by using the facile solution cast process. In detail, 25 wt % of solid pellets of PVDF are dissolved in DMF under constant stirring at 170 °C for 2 h. Simultaneously, SnO_2_ nanopowder is dispersed in a DMF solution using ultrasonication for 2 h. The SnO_2_-DMF mixture is then added to the PVDF solution, and the mixture is continuously further stirred at 60 °C for 4 h. The resulting PSO solution is cast onto a glass Petri dish, and the DMF is evaporated by heating at 80 °C for 8 h, producing PSO nanocomposite films with concentrations of 0.5 wt %, 1.0 wt %, 2.0, 3, and 4.0 wt %. The conducting core comprises conducting polyethylene (cPE), prepared by consolidating the cPE film using a PTFE tube at 220 °C in the consolidation chamber. The PVDF nanocomposite film is then wrapped around the cPE core to create the active tribonegative layer in the preform, resulting in an ∼16 mm diameter. To ensure uniform thermal drawing, a sacrificial PC layer is rolled over the PVDF nanocomposite layer. The preform diameter measures ∼18 mm without the PC layer and 22 mm with it. The assembled preform was initially annealed under a vacuum at 90 °C for 24 h. Subsequently, a three-step consolidation treatment was carried out in a tube furnace under a continuous vacuum: (i) annealing at 150 °C for 2 h, (ii) consolidation at 175 °C for 45 min, and (iii) cooling to ambient temperature. Fiber drawing is conducted by using a custom thermal draw tower at 254 °C while maintaining controlled tension via capstone pulling. This ensures a uniform diameter and continuous production of fibers that are tens of meters long. The sacrificial PC layer is then dissolved in acetone for 10 min, yielding SnO_2_ reinforced PVDF TENG fiber. We rolled the nanocomposite films onto a consolidated cPE cylindrical rod and added a PC layer on top before consolidating the preform again. This sacrificial layer stabilized the structure during the thermal drawing process. Using a thermal drawing technique, we produced fibers of significant length by feeding the preform into a furnace at a controlled speed (*v*_feed_), maintaining a temperature above the polymers’ glass transition temperature (*T*_g_), and pulling the fibers with a capstan at a defined speed (*v*_draw_) as given in eq 1. Thermal drawing enabled the codrawing of different materials, but it required selecting materials with compatible glass transition temperatures and similar complex shear viscosity (η*) at the drawing temperature. Alongside viscosity, we carefully analyzed other rheological properties, including the storage modulus (*G*′), loss modulus (*G*″), and loss factor (tan δ). At the drawing temperature, the viscous component (loss modulus) needs to dominate the elastic component (storage modulus) to achieve a stable drawing process. Incorporating SnO_2_ significantly increased the storage modulus and complex viscosity of PVDF, which limited SnO_2_ composition to 5 wt % in this study to ensure successful fiber fabrication.

### 4.4 Material Characterization

The structural and morphological analyses of the prepared materials were characterized using an environment scanning electron microscope (ESEM, FEI) and X-ray diffraction (XRD, Rigaku) with Cu Kα radiation (λ = 1.5406 Å). A Fourier transform infrared (FT-IR) spectrometer (Bruker ALPHA Platinum-ATR FTIR) was employed to analyze the molecular structure, while an X-ray photoelectron spectrometer (XPS, NEXSA, Thermo Scientific) was utilized to investigate the surface chemistry of the materials. The microstructure was examined by using a transmission electron microscope (TEM, JEOL JEM-1400 Plus, JEOL, Peabody, MA, USA). DSC and DMA were used to understand the thermal and viscoelastic behavior of the samples.

### 4.5 Electrical Characterization

A 10 cm-long sample with a 0.5 cm exposed cPE electrode section was utilized for electrical characterization. The open-circuit voltage (*V*_oc_) was measured by using a Tektronix TBS2000B Series oscilloscope. For the short-circuit current (*I*_sc_), a combination of an oscilloscope and a Stanford Research Systems SR570 low-noise current preamplifier was employed. A single-phase induction motor with linear motion lever was used to tap the fibers with 7 cm diameters tapping flat surface. Also, another system made up of NEMA 17 stepper motor, controlled via Arduino, was used to perform cyclic tapping for sensitivity measurement. The motor was programmed to operate in a sinusoidal motion with adjustable frequencies. We have also used the electrochemical capacitor to charge them with the fibers. Impedance and dielectric properties were measured using the Keysight LCR meter.

## Supplementary Material













## Data Availability

All data needed to evaluate the conclusions in the paper are present in the paper and/or the .

## Notes

Preform and fiber morphology (SEM, elemental mapping); FTIR, XRD, and XPS analyses of PVDF and PVDF/SnO_2_ nanocomposites; detailed experimental setups for TENG measurements and motor control systems; theoretical equations for *V*_oc_, *I*_sc_, and *Q*_sc_; durability and stability tests over cyclic operation; rectifier circuits and energy storage demonstrations (LED powering and capacitor charging); flexibility and bending tests; equivalent circuit fitting parameters from impedance spectroscopy; comparative performance table of fiber-based TENGs (PDF)
Supplementary videos demonstrating device operation (MP4-1, MP4-2, MP4-3, MP4-5, MP4-6)
