# Supplementary material for: An Electromechanical Power Law in Sustainable Thermally Drawn Triboelectric Nanocomposite Fibers for Sensing in Continuum Robots
Source: ACS Appl Mater Interfaces. 2026 Apr 1;18(14):20479–98. doi: 10.1021/acsami.6c00217 (PMC13088032; doi:10.1021/acsami.6c00217)
Supplement: Supplementary file 1 [file am6c00217_si_001.pdf]

Supplementary Data

An Electromechanical Power Law in Sustainable Thermally Drawn Triboelectric Nanocomposite Fibers for sensing in Continuum robots

Vishwa Pratap Singh[1], Nurbolat Issatayev[2], Syed Zubair Hussain[1], Yersaiyn Busharov[2], Gulnur Kalimuldina*,[2], Mustafa Ordu*,[1]

[1]UNAM - National Nanotechnology Research Center and Institute of Materials Science and Nanotechnology, Bilkent University, Ankara 06800, Türkiye

[2]Department of Mechanical and Aerospace Engineering, School of Engineering and Digital Sciences, Nazarbayev University, Astana 010000, Kazakhstan

E-mail : ordu@unam.bilkent.edu.tr, gkalimuldina@nu.edu.kz

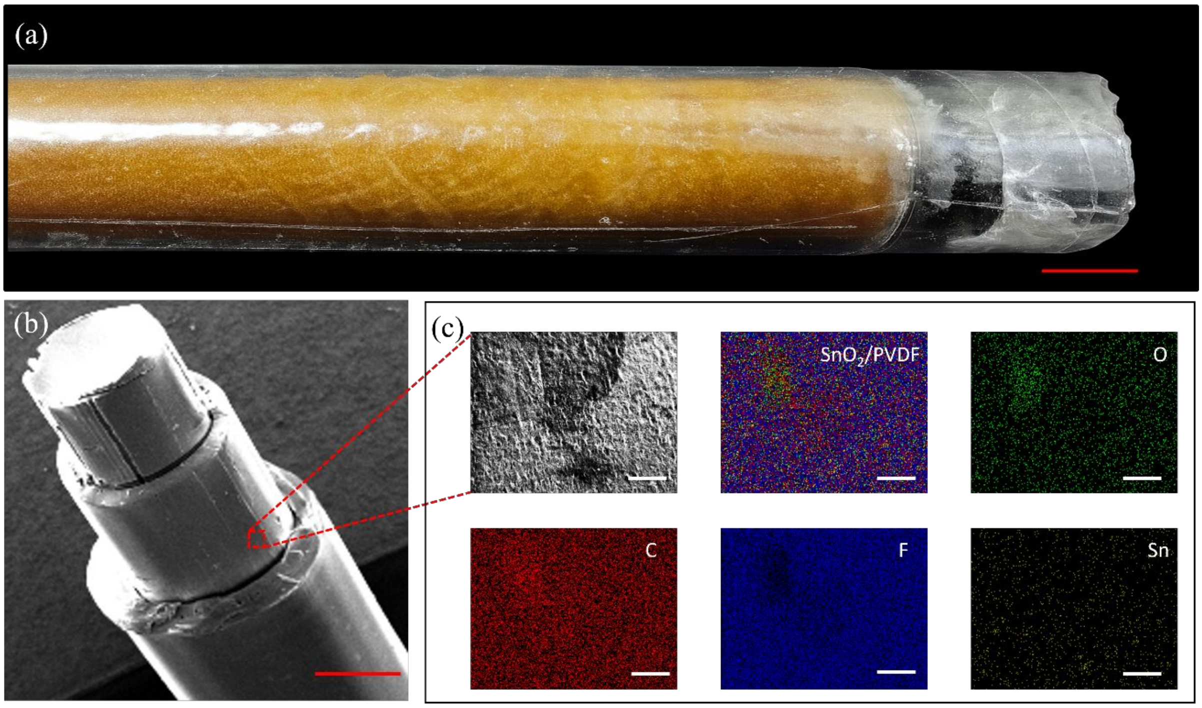

Figure S1. (a) Prepared preform (scale bar: 90 mm). (b) Perspective image of the PSO fiber (scale bar: 200 μm) with (c) the zoomed area for the elemental mapping showing the SnO_2 distribution at 4.0 % reinforcement (scale bar: 50 μm).

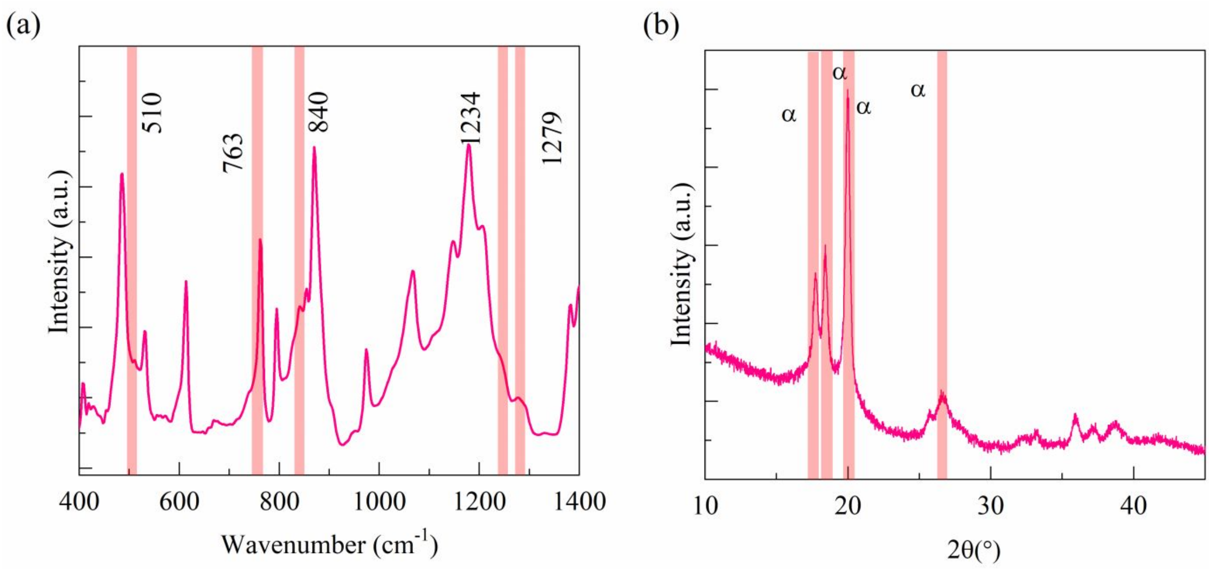

Figure S2. (a) FTIR spectrum of PVDF film with several characteristic absorption peaks of α- and β-phases at 510 cm^{-1}, 763 cm^{-1}, 840 cm^{-1}, 1234 cm^{-1}, and 1279 cm^{-1}. (b) XRD pattern of PVDF film with diffraction peaks corresponding to the α-phase.

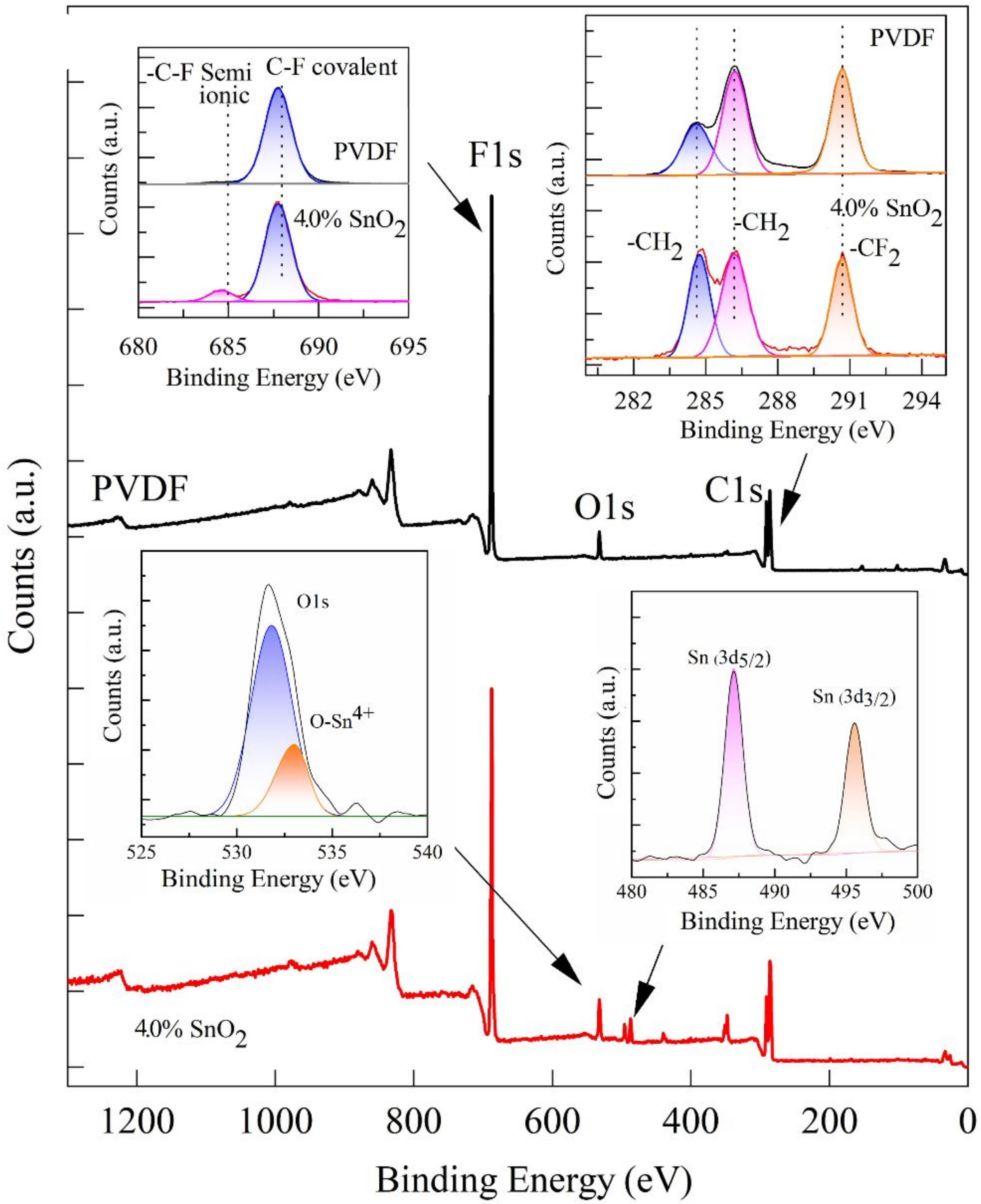

Figure S3. XPS of pristine PVDF and SnO_2 integrated PVDF.

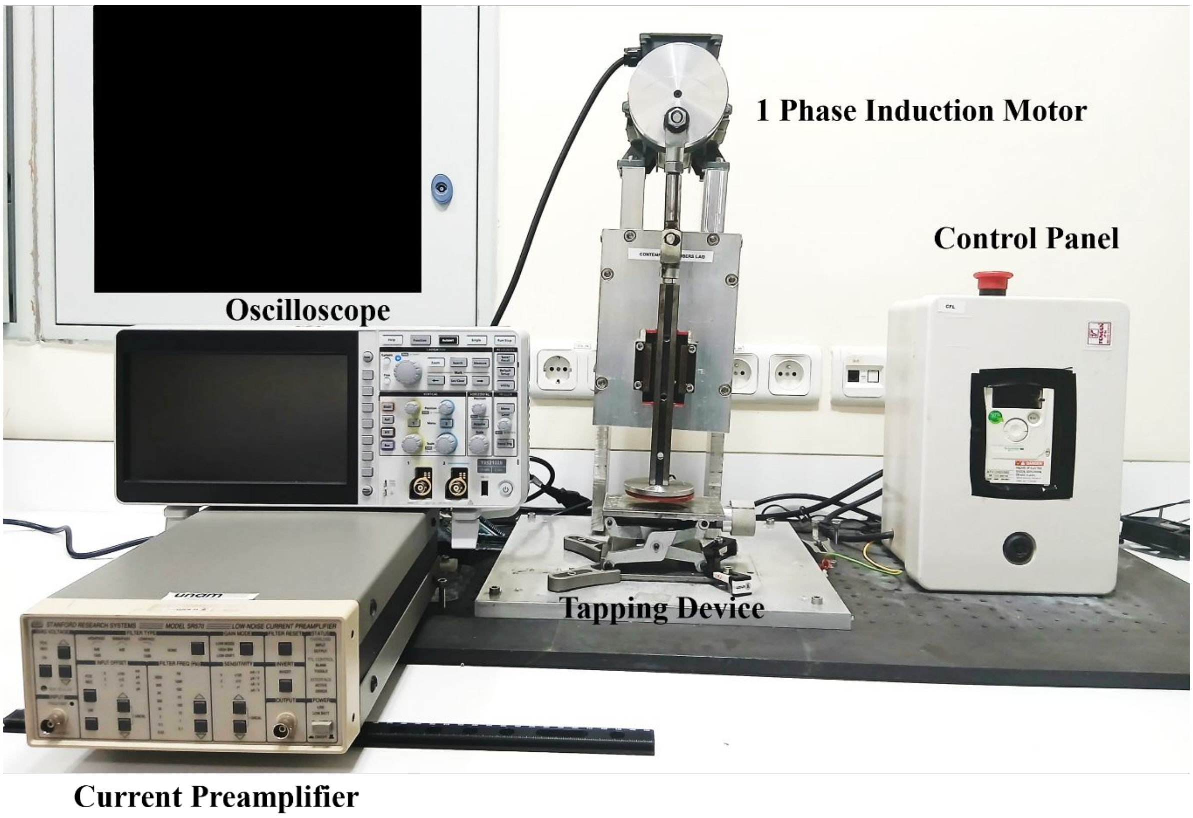

Figure S4. Experimental setup for fiber-based triboelectric nanogenerators (TENG) measurements, consisting of a custom-made tapping device driven by a single-phase induction motor, a control panel, an oscilloscope, and a low-noise current preamplifier.

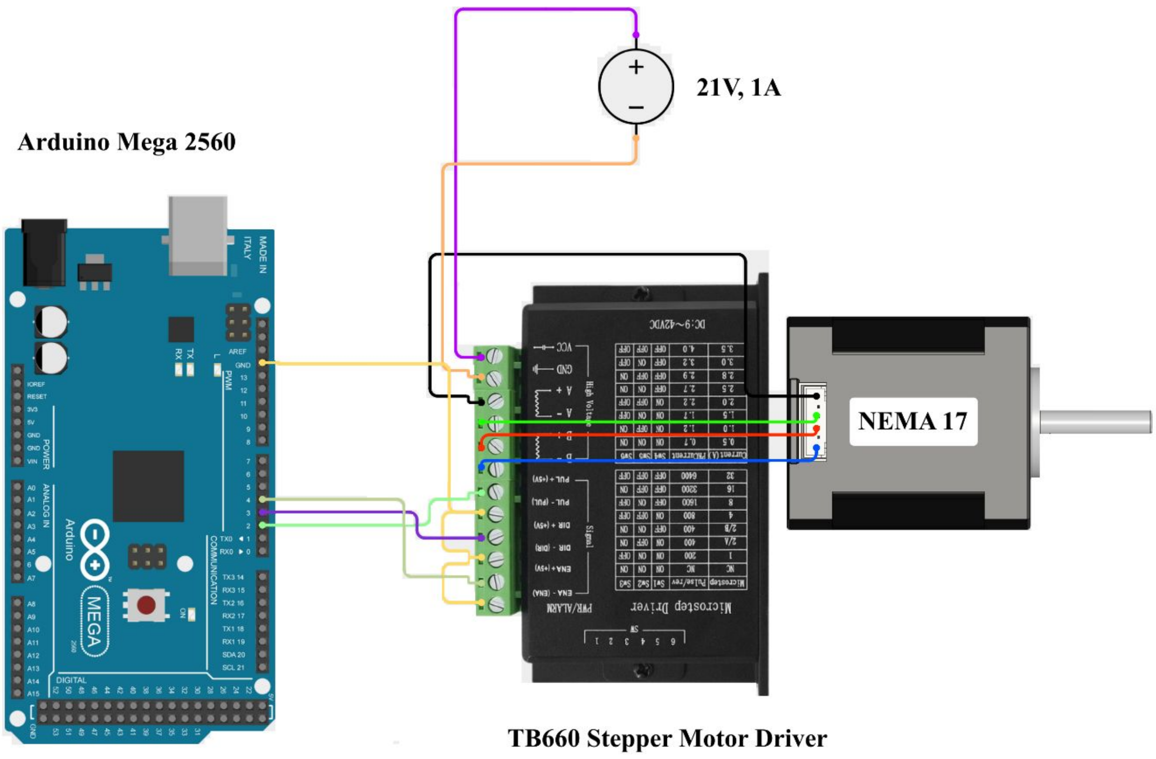

Figure S5. Circuit diagram of the stepper motor control setup showing the connection between Arduino Mega 2560, TB660 stepper motor driver, and NEMA 17 motor powered by a 21 V and 1 A DC supply.

The following equations give the open circuit voltage (V_{oc}), short circuit current (I_{sc}), and short circuit charge transfer (Q_{sc}) by this cyclic contact separation process:

$$V_{oc} = \sigma S_2 C_0 \tag{S1}$$

$$I_{sc} = \frac{dQ_{sc}}{dt} \tag{S2}$$

$$Q_{sc} = \sigma S_2 \tag{S3}$$

where, σ, S and C_0 represent the average surface charge density triboelectric material, contact area of the device, and is capacitance using vacuum as the dielectric, respectively. Eqs. (S4) and (S5) reveal the dependency of the triboelectric output on the contact area and surface charge density. Moreover, the charge density of the surface depends on the capacitance and the dielectric property of the functional surface and is expressed as follows:

$$\sigma = \frac{CV}{S} = \varepsilon_0 \varepsilon_r \frac{V}{t} \tag{S4}$$

$$C = \varepsilon_0 \varepsilon_r \frac{S}{t} \tag{S5}$$

Here, C denotes the capacitance, ε_0 the permittivity of free space, ε_r the relative permittivity of the material, V the fiber's surface potential, and t the fiber thickness.

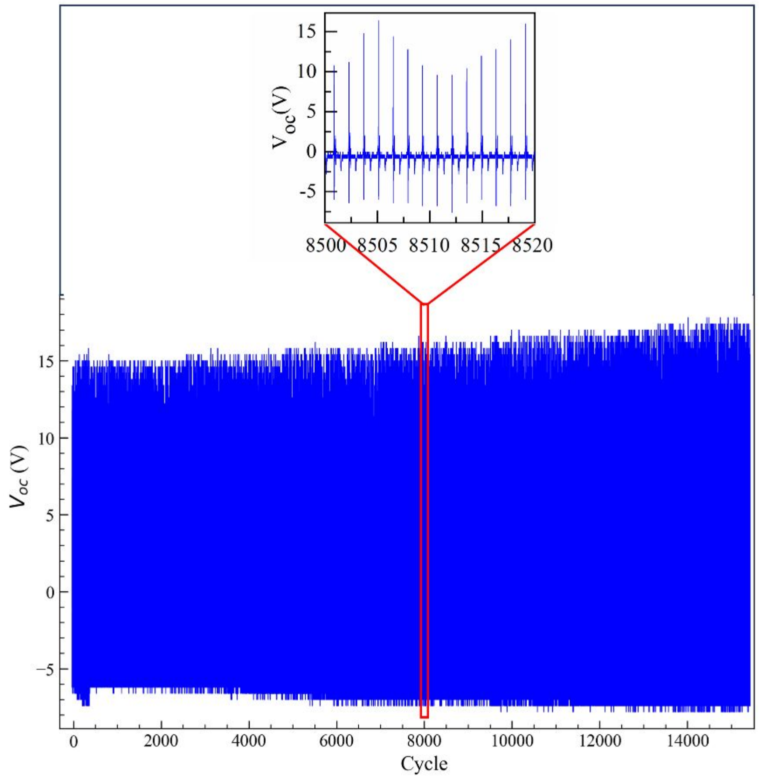

Figure S6. Durability test of the device showing stable open-circuit voltage (V_{oc}) performance over 16,000 cycles (-800 to 800 seconds with 10 Hz). The inset highlights the consistent output waveform between 8,500–8,520 cycles.

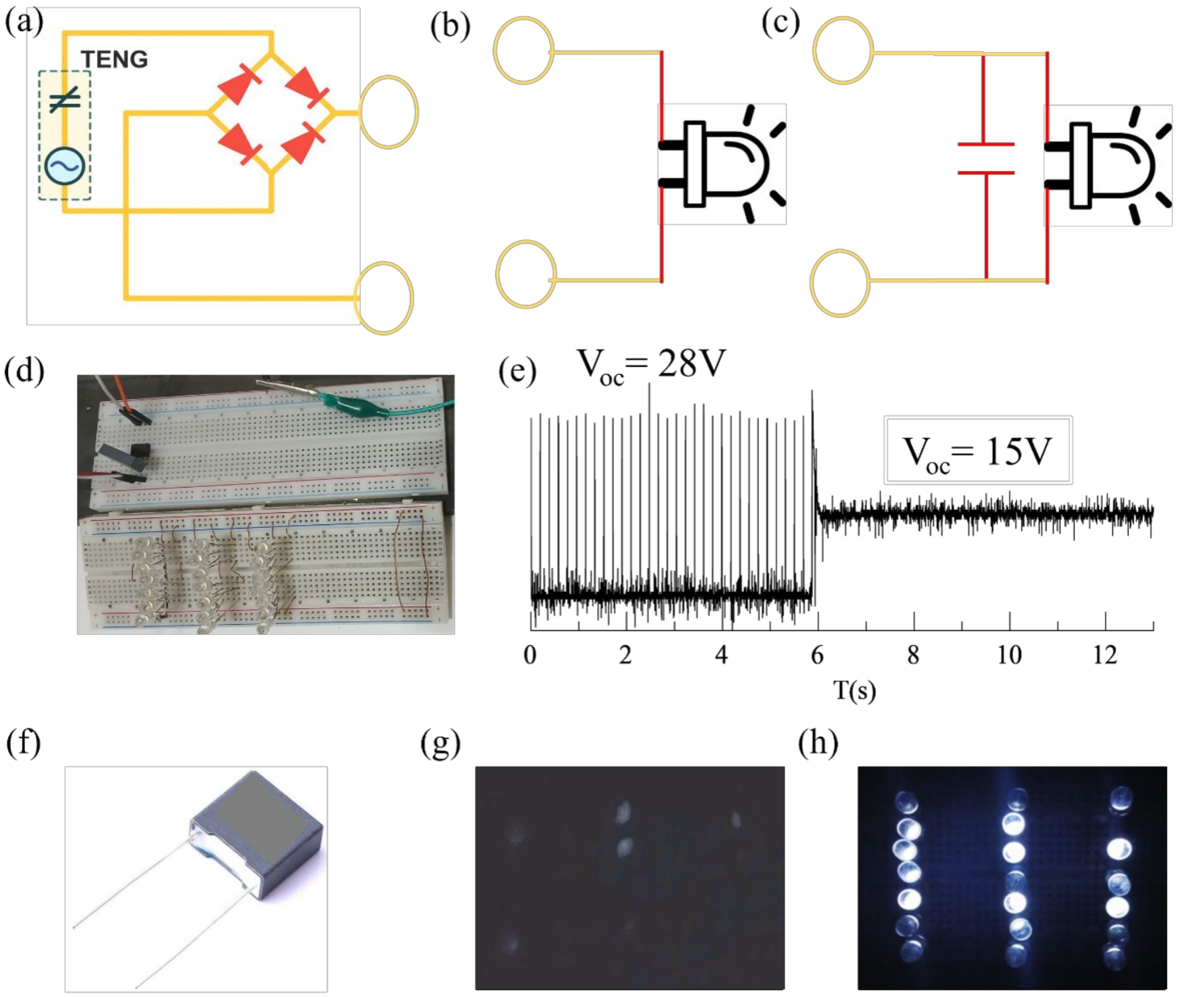

Figure S7. (a) Schematic diagram of the rectifier circuit connected to the TENG output for AC-to-DC conversion. (b) Direct powering of LEDs using the TENG output. (c) Capacitor-assisted circuit for continuous LED illumination. (d) Experimental setup showing the rectifier and LED array on the breadboard. (e) Open-circuit voltage of the TENG before and after capacitor connection in the circuit. (f) Film capacitor employed to obtain a smooth DC voltage. (g) Circuit corresponding to panel (g) demonstrating LED blinking behavior and also shown in **VideoS**4 and (h) continuous LED illumination driven by the TENG before and after capacitor connection also shown in **VideoS5**, respectively.

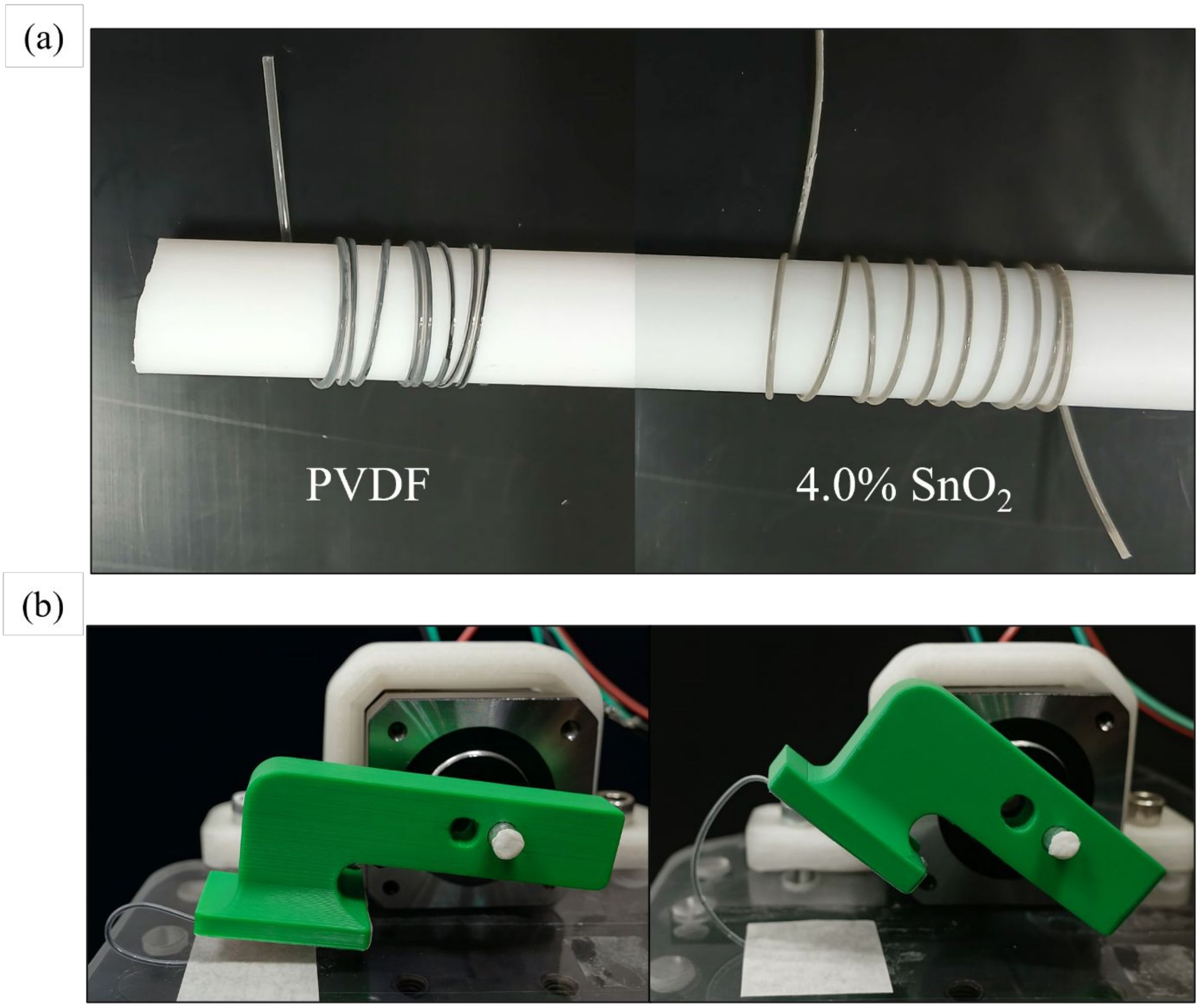

Figure S8. Flexibility comparison of pristine PVDF and 4.0 % SnO_2 PSO fibers by wrapping around a Polytetrafluoroethylene (PTFE) rod and (b) stepper motor used for bending test.

Table S1. Equivalent circuit parameters obtained from Nyquist plot fitting of PVDF/SnO_2 nanocomposite fibers.

SnO_2 (wt%)	RMSE	T_{CPE1} (F·s^(p^{-1}))	R (Ω)	R_1 (Ω)	p_{CPE1}	p_{CPE2}	T_{CPE2} (F·s^(p^{-1}))
0	1.57×10^7	2.87×10^{-12}	55.0	1.00×10^{12}	0.97	0.98	1.12×10^{-8}
0.5	2.03×10^6	5.56×10^{-12}	55.0	1.00×10^{12}	0.97	0.98	2.35×10^{-8}
1	6.27×10^6	6.52×10^{-12}	55.0	1.00×10^{12}	0.97	0.99	8.26×10^{-8}
2	8.80×10^6	7.64×10^{-12}	50.0	5.50×10^{12}	0.97	0.96	2.52×10^{-8}
3	5.01×10^6	8.43×10^{-12}	50.0	5.50×10^{12}	0.97	0.97	3.14×10^{-8}
4	6.90×10^6	1.44×10^{-11}	55.7	1.00×10^{12}	0.96	0.99	1.39×10^{-8}

Table S2. Comparison of fiber-based TENG performance from this work and reported literature.

Structure	Voltage (V_{oc}) and Current (I_{sc})	Power Output	Load Resistance	Reference
SnO_2/PVDF fiber-based TENG	37.2 V and 36.25 µA	32.11 µW, 243 mW m^{-2}	9 MΩ	**This work**
MXene/PVDF fiber-based TENG	30 V and 29.6 µA	24.5 µW, 40.8 mW m^{-2}	8 MΩ	[1]
Acrylic/Al/PTFE-based double mode TENG	45V and 15 µA	3 mW, 9.8 W m^{-2}	400 MΩ	[2]

Triboelectrification enhancement effect (TEE)/opposite-charge-based TENG	180 V and 2.5 µA	20.6 W m^{-3}	--	3
Polylactide (PLA)/steel spring-based TENG	120 V and 0.87 µA	242.4 mW m^{-2}	500 MΩ	4
PTFE/Cu/polyimide (PI) multilayer circular stacked TENG	454.8 V and 11.2 µA	9.7 mW, 2.16 W m^{-2}	3 MΩ	5
PTFE/Cu electrode-grounded flow-based TENG	102 V and 22 µA	1.9 W m^{-2}	16 MΩ	6
Polyamide (PA66)/PTFE yarn-based textile-electrode TENG	~ 232 V and 6.8 µA	66.13 mW m^{-2}	10 MΩ	7
All-printed flexible CS-mode TENG	60 V and ~2 nA	70 µW, 34 mW m^{-2}	12.3 MΩ	8
Ni-coated sponge/flexible dielectric tubular TENG	196.4 V and 30.2 µA	112.5 mW m^{-2}	50 MΩ	9
Hierarchically textured (TH) organic–inorganic hybrid Indium tin	80 V and 12.5 µA	824 mW m^{-2}	1 MΩ	10

oxide (ITO)/PET)-based THTENG				
CNT/nylon core-P(VDF-TrFE)/Ag fiber-Ecoflex embedded TENG	56.8 V and ~0.58 μA	95.5 µW m^{-1}	100 MΩ	[11]

VideoS1: Flexibility

VideoS2: Under contact-separation mode along the Y-axis with the baloon

VideoS3 Output voltage under swinging-over mode along the Z-axis

VideoS4: LED blinking without Capacitor

VideoS5: LEDs with Capacitor

References

(1) Hasan, M. M.; Sadeque, M. S. B.; Albasar, I.; Pecenek, H.; Dokan, F. K.; Onses, M. S.; Ordu, M. Scalable Fabrication of MXene-PVDF Nanocomposite Triboelectric Fibers via Thermal Drawing. *Small* **2023**, *19* (6), 2206107. https://doi.org/10.1002/smll.202206107.

(2) Shan, C.; He, W.; Wu, H.; Fu, S.; Li, G.; Du, Y.; Wang, J.; Mu, Q.; Guo, H.; Liu, B.; Hu, C. Efficiently Utilizing Shallow and Deep Trapped Charges on Polyester Fiber Cloth Surface by Double Working Mode Design for High Output and Durability TENG. *Nano Energy* **2022**, *104*, 107968. https://doi.org/10.1016/j.nanoen.2022.107968.

(3) Yu, Y.; Li, H.; Zhang, X.; Gao, Q.; Yang, B.; Wang, Z. L.; Cheng, T. Substantially Boosting Performance of Triboelectric Nanogenerators via a Triboelectrification Enhancement Effect. *Joule* **2024**, *8* (6), 1855–1868. https://doi.org/10.1016/j.joule.2024.04.013.

(4) Zhao, Y.; Feng, Y.; Gao, Q.; Li, H.; Guo, X.; Wang, J.; Wang, X.; Dong, L.; Yu, Y.; Wang, Z. L.; Cheng, T. Boosting Output Performance of Triboelectric Nanogenerator via Interface Self-Regulation Strategy. *Research* **2025**, *8*, 0906. https://doi.org/10.34133/research.0906.

(5) Shen, F.; Zhang, Q.; Guo, H.; Cao, C.; Gong, Y.; Wang, J.; Peng, Y.; Li, Z. Investigation of Power Density Amplification in Stacked Triboelectric Nanogenerators. *ENERGY Environ. Mater.* **2024**, *7* (5), e12697. https://doi.org/10.1002/eem2.12697.
(6) Munirathinam, K.; Shanmugasundaram, A.; Jeong, Y.-J.; Kim, J.-Y.; Lee, D.-W. High-Performance Triboelectric Nanogenerator Powered by Flowing Water for Self-Powered Wireless Sensor Platform. *Chem. Eng. J.* **2025**, *505*, 159106. https://doi.org/10.1016/j.cej.2024.159106.
(7) Xu, F.; Dong, S.; Liu, G.; Pan, C.; Guo, Z. H.; Guo, W.; Li, L.; Liu, Y.; Zhang, C.; Pu, X.; Wang, Z. L. Scalable Fabrication of Stretchable and Washable Textile Triboelectric Nanogenerators as Constant Power Sources for Wearable Electronics. *Nano Energy* **2021**, *88*, 106247. https://doi.org/10.1016/j.nanoen.2021.106247.
(8) Liu, G.; Gao, Y.; Xu, S.; Bu, T.; Xie, Y.; Xu, C.; Zhou, H.; Qi, Y.; Zhang, C. ONE-STOP Fabrication of Triboelectric Nanogenerator Based on 3D Printing. *EcoMat* **2021**, *3* (5), e12130. https://doi.org/10.1002/eom2.12130.
(9) Liu, G. X.; Li, W. J.; Liu, W. B.; Bu, T. Z.; Guo, T.; Jiang, D. D.; Zhao, J. Q.; Xi, F. B.; Hu, W. G.; Zhang, C. Soft Tubular Triboelectric Nanogenerator for Biomechanical Energy Harvesting. *Adv. Sustain. Syst.* **2018**, *2* (12), 1800081. https://doi.org/10.1002/adsu.201800081.
(10) Lee, H.; Lee, H. E.; Wang, H. S.; Kang, S.; Lee, D.; Kim, Y. H.; Shin, J. H.; Lim, Y.; Lee, K. J.; Bae, B. Hierarchically Surface-Textured Ultrastable Hybrid Film for Large-Scale Triboelectric Nanogenerators. *Adv. Funct. Mater.* **2020**, *30* (49), 2005610. https://doi.org/10.1002/adfm.202005610.
(11) Zhang, Y.; Li, Y.; Cheng, R.; Shen, S.; Yi, J.; Peng, X.; Ning, C.; Dong, K.; Wang, Z. L. Underwater Monitoring Networks Based on Cable-Structured Triboelectric Nanogenerators. *Research* **2022**, *2022*, 2022/9809406. https://doi.org/10.34133/2022/9809406.
